# Lysine and the Na^+^/K^+^ Selectivity in Mammalian Voltage-Gated Sodium Channels

**DOI:** 10.1371/journal.pone.0162413

**Published:** 2016-09-01

**Authors:** Yang Li, Huihui Liu, Mengdie Xia, Haipeng Gong

**Affiliations:** MOE Key Laboratory of Bioinformatics, School of Life Sciences, Tsinghua University, Beijing 100084, China; Russian Academy of Medical Sciences, RUSSIAN FEDERATION

## Abstract

Voltage-gated sodium (Na_v_) channels are critical in the generation and transmission of neuronal signals in mammals. The crystal structures of several prokaryotic Na_v_ channels determined in recent years inspire the mechanistic studies on their selection upon the permeable cations (especially between Na^+^ and K^+^ ions), a property that is proposed to be mainly determined by residues in the selectivity filter. However, the mechanism of cation selection in mammalian Na_v_ channels lacks direct explanation at atomic level due to the difference in amino acid sequences between mammalian and prokaryotic Na_v_ homologues, especially at the constriction site where the DEKA motif has been identified to determine the Na^+^/K^+^ selectivity in mammalian Na_v_ channels but is completely absent in the prokaryotic counterparts. Among the DEKA residues, Lys is of the most importance since its mutation to Arg abolishes the Na^+^/K^+^ selectivity. In this work, we modeled the pore domain of mammalian Na_v_ channels by mutating the four residues at the constriction site of a prokaryotic Na_v_ channel (Na_v_Rh) to DEKA, and then mechanistically investigated the contribution of Lys in cation selection using molecular dynamics simulations. The DERA mutant was generated as a comparison to understand the loss of ion selectivity caused by the K-to-R mutation. Simulations and free energy calculations on the mutants indicate that Lys facilitates Na^+^/K^+^ selection by electrostatically repelling the cation to a highly Na^+^-selective location sandwiched by the carboxylate groups of Asp and Glu at the constriction site. In contrast, the electrostatic repulsion is substantially weakened when Lys is mutated to Arg, because of two intrinsic properties of the Arg side chain: the planar geometric design and the sparse charge distribution of the guanidine group.

## Introduction

Voltage-gated sodium (Na_v_) channels play critical roles in electrical signaling in the nervous system, heart and muscle of mammals, and their dysfunction causes numerous diseases, including epilepsy, migraine and cardiac arrhythmias [1–3]. Similar to voltage-gated potassium (K_v_) channels [4], prokaryotic Na_v_ channels are comprised of four identical subunits, an architecture that greatly simplifies their structural determination. In recent years, a series of crystal structures of prokaryotic Na_v_ channels were successively solved [5–7], laying structural foundation for the study on channelopathies [8, 9]. In mammalian Na_v_ channels, the corresponding structural units are, however, linearly connected in a single polypeptide [1, 2], which complicates and retards their crystallographic studies.

Ion selectivity is an essential characteristic in all types of voltage-gated cation channels [10]. In mammalian Na_v_ channels, K^+^ ions must be selectively excluded, because otherwise the rising phase of action potential would be substantially suppressed due to the simultaneously inward flow of extracellular Na^+^ ions and outward flow of cytoplasmic K^+^ ions upon channel opening. According to mutational analyses, four highly conserved residues (Asp, Glu, Lys and Ala, also called DEKA motif) within the selectivity filter (SF) of the pore domain (PD) [11–14] are primarily responsible for the Na^+^/K^+^ selectivity in mammalian Na_v_ channels. In some available prokaryotic Na_v_ structures, four Glu residues are present at the positions of the DEKA motif, composing the EEEE ring (also called Site_HFS_ for high field strength). Since the EEEE ring coincides with the geometrically most constricted region in the SF, the DEKA motif (or DEKA ring) is sometimes denoted as the constriction site. Among the DEKA residues, Lys is of the most importance, because its mutations to Ala or even Arg completely abolish the Na^+^/K^+^ selectivity of mammalian Na_v_ channels [12]. Unfortunately, the molecular mechanism of Na^+^/K^+^ selectivity in mammalian Na_v_ channels, especially the role of Lys, remains elusive due to the lack of available crystal structures, despite a few pioneering theoretical studies performed in the absence of atomic details [15–22].

Complementary to traditional experimental methods, molecular dynamics (MD) simulations can illustrate atomic-level intuitive dynamics of macromolecules, and have therefore become critical tools in the investigation of a wide range of chemical and biological systems [23, 24]. Immediately after the structural determination of the first Na_v_ channel (Na_v_Ab), several simulation studies have been initiated on this bacterial channel to study the binding sites and permeation mechanisms of cations [25–33]. These works not only confirmed the presence of four cation binding sites (IonEX, SiteHFS, SiteCEN and SiteIN) in the SF as proposed from the crystal structure [25, 34], but also suggested considerable structural variations when cations bind at these sites [31, 32]. Therefore, the definition of cation binding sites simply based on their relative positions from the geometric center of SF in the static crystal structure cannot adequately describe the ion binding and permeation. In addition, the protonation states of the Glu residues in the EEEE ring are also indicated to affect the cation binding pattern in the SF of Na_v_Ab [32, 33].

**Fig 1:**
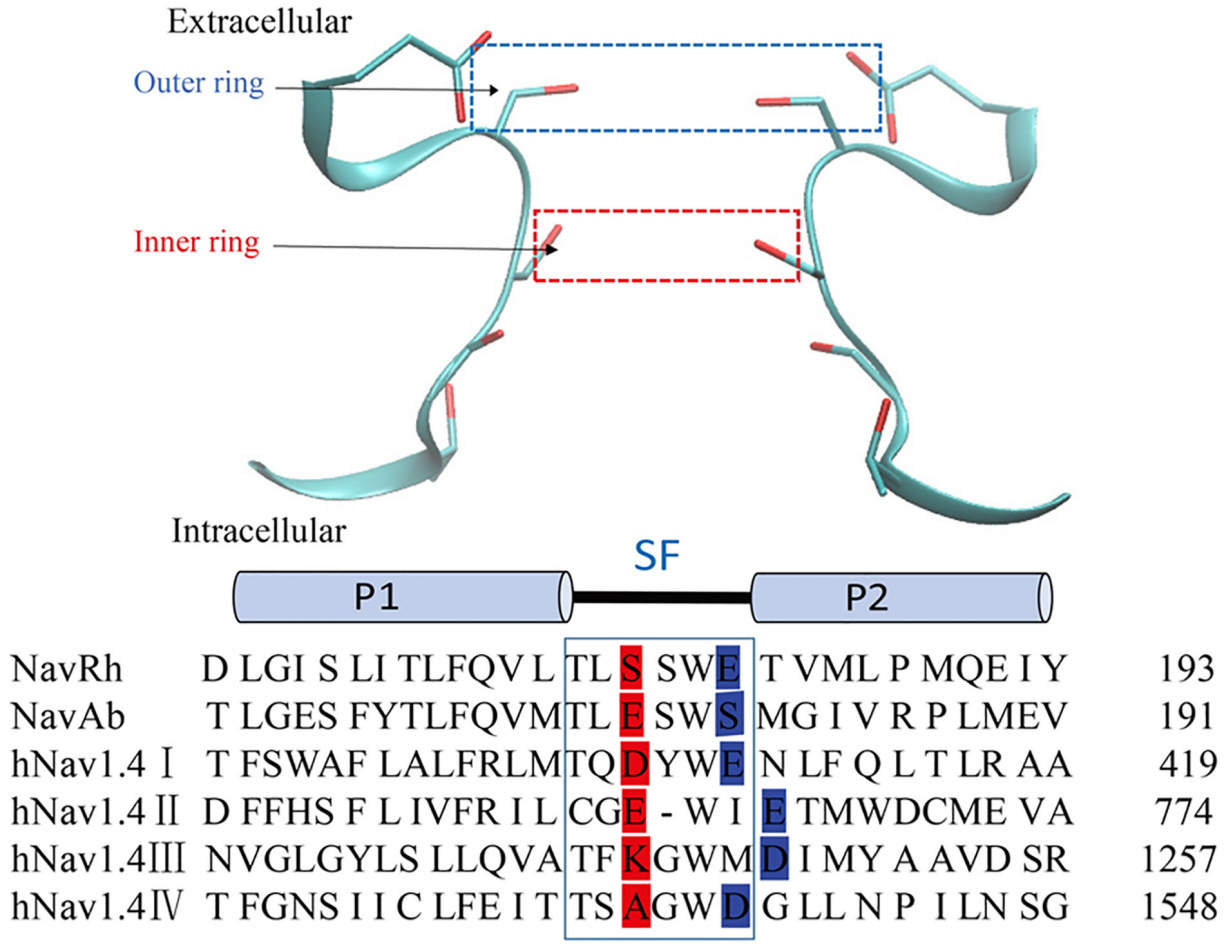
The structural representation and sequence alignment for the SFs of Na_v_ channels. In the structural representation (upper panel), the Na_v_Rh crystal structure is shown as an example, where only two of the four chains (A and C) are represented in cartoons for clarity. In the sequence alignment (lower panel), the sequences for two prokaryotic channels (Na_v_Rh and Na_v_Ab) as well as the four domains of one mammalian channel (human Na_v_1.4) are compared side-by-side, with the P-loop of the SF region highlighted by a blue frame. Two rings of residues are highly conserved in mammalian Na_v_ channels: the inner ring (shaded in red) and the outer ring (shaded in blue). The positions of these two rings are labeled by dotted frames colored in red and blue respectively, in the structural representation (upper panel).

Despite the lack of crystal structures, the structural information of mammalian Na_v_ channels could be inferred from their prokaryotic homologues, due to their considerable sequence similarity, especially in the SF [35–37]. So far, there have been a few attempts to study the toxin binding pattern using homology models of mammalian Na_v_1.4 channel that were constructed from the Na_v_Ab crystal structure [38–42]. These researches suggest the presence of a common toxin binding motif, the EEDD ring, which is located at the extracellular entrance of the SF, three residues away from the DEKA ring [32, 41] (Table A in S1 File and Fig 1). Moreover, the DEKA ring (or the inner ring) and EEDD ring (or the outer ring) are both highly conserved in the mammalian Na_v_ channels [43], and have been identified as responsible for the Na^+^/K^+^ selectivity and the efficient permeation of Na^+^ ions respectively. Notably, prokaryotic channels Na_v_Ab and Na_v_Rh exhibit an EEEE ring at the loci equivalent to the inner and outer rings respectively [6, 24, 34] (Table A in S1 File and Fig 1). In a recent work, MD simulations were conducted on a model structure of Na_v_1.4 channel to investigate the ion permeation in mammalian Na_v_ channels [44]. However, their simulations showed constitutively stable hydrogen bonding interactions between Lys in the DEKA ring and the nearby Ser even before the cation occupancy [44], a phenomenon that is intuitively unlikely to occur considering the high flexibility of Lys side chain. In addition, this observation is inconsistent with numerous previous theoretical studies [15, 16], which proposed that the flexible side chain of Lys is more likely to form hydrogen bonds with the opposing Asp/Glu in the DEKA ring in the absence of cations. Therefore, the Na^+^/K^+^ selectivity in mammalian Na_v_ channels still awaits more simulation studies.

In our previous work, we created a simple structural model of the mammalian Na_v_ channel by mutating Ser residues at the constriction site of Na_v_Rh to DEKA and then investigated the mechanism of Na^+^/K^+^ selectivity using MD simulations [45]. Due to the very limited mutational interference introduced, the model generated in this manner exhibited highly flexible Lys side-chain conformation, thus avoiding the constitutive entrapment of this residue by any hydrogen bonding partners. We observed that the SF of the mutant channel is open only when Lys forms favorable interactions with acidic residues in the outer ring of Na_v_Rh (see Table A in S1 File). According to our calculations, Lys facilitates the cation selection by repelling the cation to pass through a highly Na^+^-preferred sub-location sandwiched by the carboxylate groups of Asp and Glu at the constriction site [45]. Although the proposition reasonably explained the Na^+^/K^+^ selectivity, a constraint between Lys and Glu in the outer ring was applied during the equilibrium simulations to guarantee quick ion permeation through the SF, which might impose unexpected influence on the protein conformation. In addition, the short (50 ns) equilibrium simulations were insufficient for reliable structural investigation on the cation binding modes at the constriction site. In the present work, we extended our previous research on the mechanism of Na^+^/K^+^ selectivity using long and constraint-free equilibrium simulations. More importantly, the disruption of selectivity when Lys is mutated to Arg in mammalian Na_v_ channels was also mechanistically investigated through comparison of side-by-side simulations on the DEKA and DERA mutants of Na_v_Rh. The results suggest that the loss of Na^+^/K^+^ selectivity in the DERA mutant is mainly caused by weakened electrostatic repulsion from the Arg side chain. On one hand, unlike the amino group of Lys side chain, the planar guanidine group in the Arg side chain enables its stable bifurcate interactions with the carboxylate groups of acidic residues in the outer ring, which then attracts the positively charged Arg side chain to swing away from the center of SF pore. On the other hand, the Arg side chain has a sparser charge distribution than Lys. The two factors jointly weaken the electrostatic repulsion exerted on the cations bound at the constriction site, which therefore allows K^+^-preferred cation binding modes.

## Methods

### Model definition and basic simulation parameters

Atomic coordinates of Na_v_Rh structure were taken from the protein data bank (PDB ID: 4DXW) and were processed in a similar way to our previous work [45]. The mutant protein structures were generated using the “Mutator” plugin of VMD 1.9.1 [46]. Before simulation, the voltage-sensing domain (VSD) was removed and the PD (residue 118–227 in all chains) was inserted into the POPC lipid bilayer (containing ~187 POPC molecules). The system was then immersed in TIP3P water (~16000 water molecules) and electrically neutralized by 150 mM XCl, where X corresponds to Na^+^ or K^+^. All simulations were conducted in an NPT ensemble using NAMD 2.9 [47], where the pressure and temperature were held at 1 atm and 310 K by the Nose-Hoover Langevin piston [48, 49] and the Langevin thermostat [49] respectively. The CHARMM36 force field [50, 51] was utilized to simulate the system with CMAP correction [52]. We used NBFIX [53] to correct the interactions between Na^+^/K^+^ and Cl^-^ ions. The van der Waals interaction was cutoff at 12 Å using a smooth switch at 10 Å. The electrostatic energy was estimated using the Particle Mesh Ewald (PME) method [54] with periodic boundary conditions (PBC) engaged. The SETTLE algorithm [55] was used to restrain all the hydrogen atoms whenever the 2fs time step was used. In total, 30 equilibrium simulations were conducted (see Table B in S1 File). The detailed simulation protocols are shown in the S1 File.

## Results and Discussion

### A linkage between the outer ring and the inner ring affects ion binding and permeation

Similar to our previous work [45], the DEKA and DERA mutants were created by mutating the Ser180 residues of the wild-type (WT) Na_v_Rh to DEKA and DERA respectively. Consistent with the previous work, short simulations on the DEKA mutant in NaCl (IDs 5–6 in Table B in S1 File) indicates that the long and flexible side chain of Lys180 (in the inner ring) may preclude the ion permeation, and that only when the side chain of Lys180 closely interacts with one carboxylate group of Glu183 residues in the outer ring, could the cation quickly enter the SF and eventually arrive at the interior binding site beneath the DEKA ring (denoted as SiteINT).

**Fig 2:**
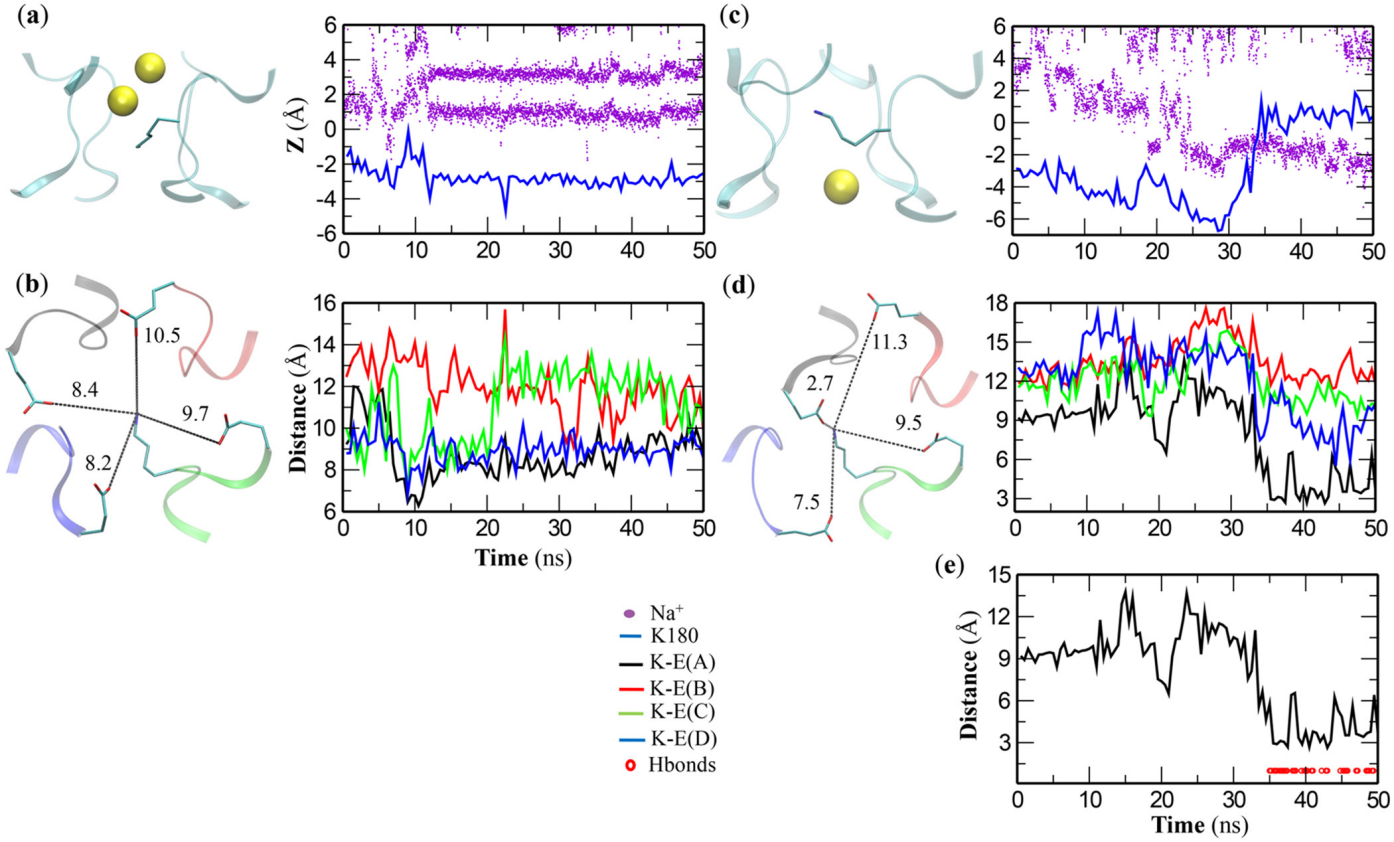
Two constraint-free short simulations on the DEKA mutant in NaCl. Na^+^ ions are represented as yellow spheres. **(a, b**) In the first simulation (ID 5 in Table B in S1 File), no Na^+^ ions (violet dots) can pass Lys180 side chain (blue line) to reach the interior Site_INT_ (**a**), and Lys180 side chain never falls within the interacting distances of any Glu183 residues (distances colored in black, red, green and blue for chain A, B, C and D respectively) (**b**). (**c-e**) In the second simulation (ID 6 in Table B in S1 File), the Na^+^ ion (violet dots) can cross Lys180 side chain (blue line) and arrive at Site_INT_ (**c**), and Lys180 side chain begins to interact with at least one of the Glu183 residues (distances colored in the same scheme) roughly at the same time. In the last 15 ns of this simulation, steady hydrogen bonds can form between Lys180 and Glu183 of chain A (**e**).

In specific, in one simulation (ID 5 in Table B in S1 File), no Na^+^ ions ever pass Lys180 and arrive at SiteINT within 50 ns, as indicated by their relative positions to the center of SF (Z) in the z-axis that is perpendicular to the membrane bilayer (Fig 2a). Coincidently, the Lys180 side chain never falls into the interaction distance of any Glu183 residues in the outer ring (Fig 2b). As a contrast, in the other simulation (ID 6 in Table B in S1 File) where one Na^+^ ion permeates across the Lys180 side chain at around 35 ns (Fig 2c), Lys180 side chain swings outwards to form hydrogen bonds with the carboxylate group of Glu183 in chain A at roughly the same time (Fig 2d and 2e).

We extracted structural snapshots from the last 15 ns trajectories of the above two simulations (IDs 5–6 in Table B in S1 File) to represent the closed and open states of the SF respectively. These structures were then plotted against two variables (R and Z) that describe the distances of the ζ-nitrogen (NZ) atom of Lys180 side chain to the center of SF projected in the xy-plane (parallel to the membrane bilayer) and z-axis respectively. As shown in Fig A in S1 File, the closed and open states could be well separated by the position of Lys180 NZ atom. Moreover, based on the best linear separation (green line in Fig A in S1 File) obtained using the support vector machine (SVM) model [56], boundaries of the 95% confidence regions for the closed and open states could be estimated (dashed lines in Fig A in S1 File). The region of the open state generally exhibits large R and Z values, indicating that Lys180 side chain has to swing outwards and move away from the center of SF pore in order to open the channel. The above estimation on confidence regions for the closed and open states is robust, since the closed SFs observed in the simulation on DEKA mutant in KCl (ID 7 in Table B in S1 File) as well as in the simulation on DERA mutant in NaCl (ID 8 in Table B in S1 File) are distributed mainly in the identified region of closed state (Table C in S1 File).

To further verify the dominant role of Lys side chain in the SF permeability, we conducted simulations of four negative control systems where Lys/Arg side chains are absent at the constriction site: WT and DEAA mutant of Na_v_Rh in NaCl and KCl respectively (IDs 1–4 in Table B in S1 File). As expected, the WT and DEAA mutant allow both Na^+^ and K^+^ ions to quickly permeate into the SF (Fig B in S1 File), consistent with the weak Na^+^/K^+^ selectivity of these channels reported in our previous works [28, 45].

Although the above results suggest that Lys180 side chain should swing outwards to interact with one of the four Glu183 residues in the outer ring so as to maintain the open SF, the specific interaction partner for Lys180 side chain needs further identification. Thus, we conducted four control simulations (IDs 9–12 in Table B in S1 File), in each of which a weak constraint with flat-bottom potential was applied in the pre-equilibration to enforce the interaction between Lys180 and one of the four Glu183 residues in the outer ring (Fig C in S1 File) but was completely released in the following 50 ns equilibrium simulation. In the simulations with pre-formed interactions between Lys180 and Glu183 residue in chain A or chain D, the SFs open quickly, and Na^+^ ions can arrive at the interior Site_INT_ within 50 ns (Figs D and E in S1 File, panel ad), although the SFs fall in the region of closed state intermittently due to the complete removal of constraints in the equilibrium simulations. Conversely, the channels with pre-formed interactions between Lys180 and Glu183 residue in chain B or chain C fail to open, because the Lys side chain still occludes the ion permeation pathway (Figs D and E in S1 File, panel bc). Although the SF may become permeable in longer time scales in these two systems, constraints applied on chain B and C are less likely to enhance the permeability of the SF than those applied on chain A and D. Consistent with these observations, according to the sequence alignment of the SFs (Table A in S1 File and Fig 1), the outer-ring acidic residues in domains II and III of mammalian Na_v_ channels shift outwards by one residue when compared to the ones in domains I and IV, implying the smaller probabilities to attract the Lys side chain (in the inner ring) of the former than of the latter.

### Ion permeation and binding in the DEKA and DERA mutants of Na_v_Rh

Since the weak constraints applied on chain A and chain D in the pre-equilibrations can guarantee arrival of the first ion at SiteINT within limited time and effectively maintain arrival of the second ion at the constriction site in the constraint-free equilibrium simulations (Figs D and E in S1 File, panel ad), we engaged this protocol in the subsequent long equilibrium simulations (IDs 13–20 in Table B in S1 File) to investigate the cation binding modes in the DEKA and DERA mutants of Na_v_Rh. Specifically, the simulations with constraints on chain A and chain D in the pre-equilibrations were denoted as **a**-system and **d**-system respectively. Notably, the constraints were removed in the 200 ns equilibrium simulations.

**Fig 3:**
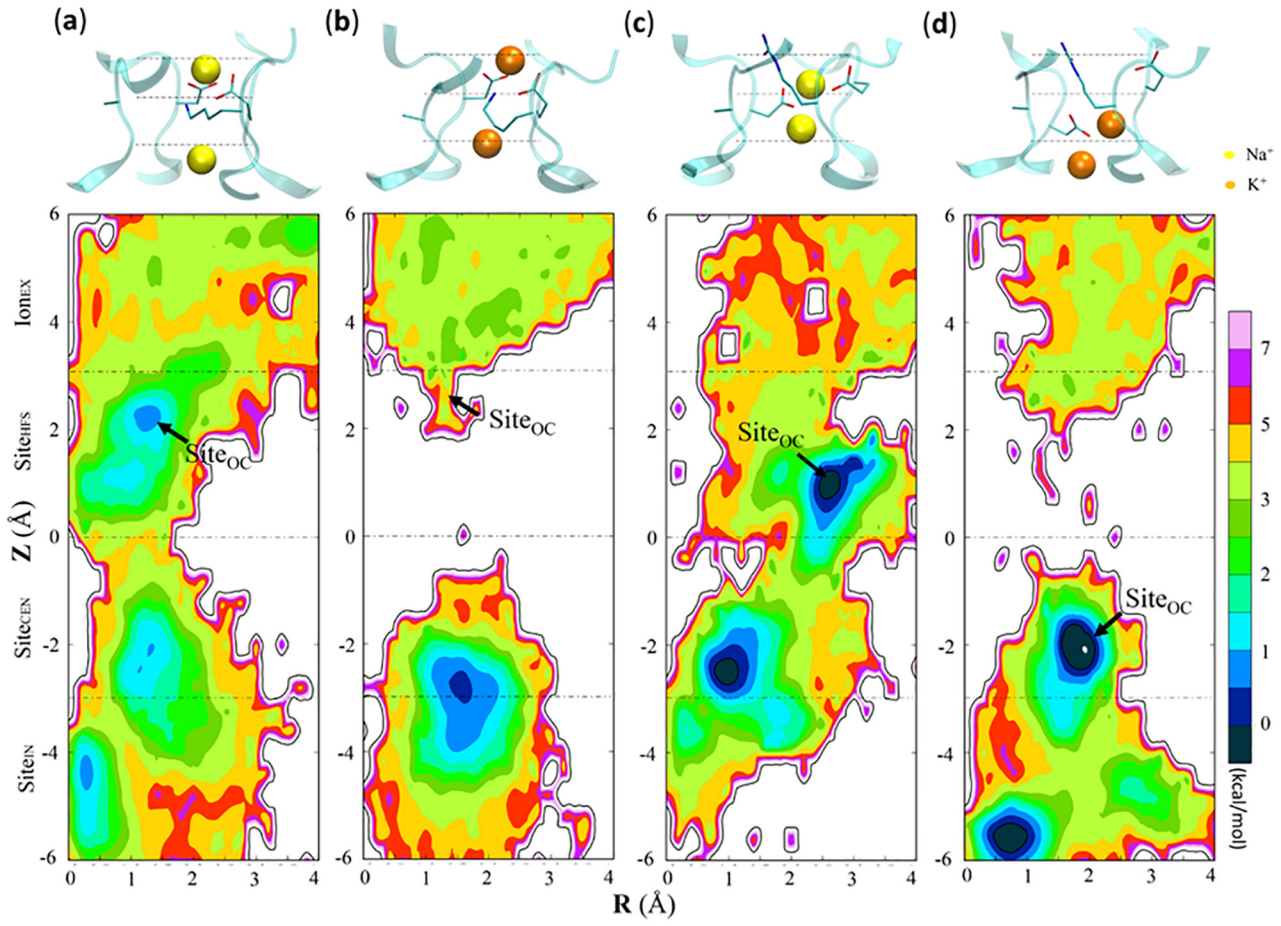
Ion binding patterns in the SF of the DEKA (a, b) and DERA (c, d) mutants of Na_v_Rh for Na^+^ (a, c) and K^+^ (b, d) ions in the a-system (IDs 13–16 in Table B in S1 File). For each map, the upper panel shows a representative structure obtained from the equilibrium simulation. The side chains of residues 180 are shown in the licorice representation for clarity. The SF is shown in the cartoon representation and colored in cyan. Na^+^ and K^+^ ions are represented as yellow and orange spheres respectively. The lower panel exhibits the 2D free energy profile (in the unit of kcal/mol) estimated from the probability density map of cations in SF that is counted from the corresponding 200 ns equilibrium simulation. The vertical axis is the relative distance of the cation to the geometric center of the SF along the z-axis, with labels denoting the binding sites (Ion_EX_, Site_HFS_, Site_CEN_ and Site_IN_) proposed from the static crystal structure of Na_v_Ab. Positions of Site_OC_ are labeled by black arrows. The horizontal axis is the distance between cation and the geometric center of the SF projected in the xy-plane.

**Fig 4:**
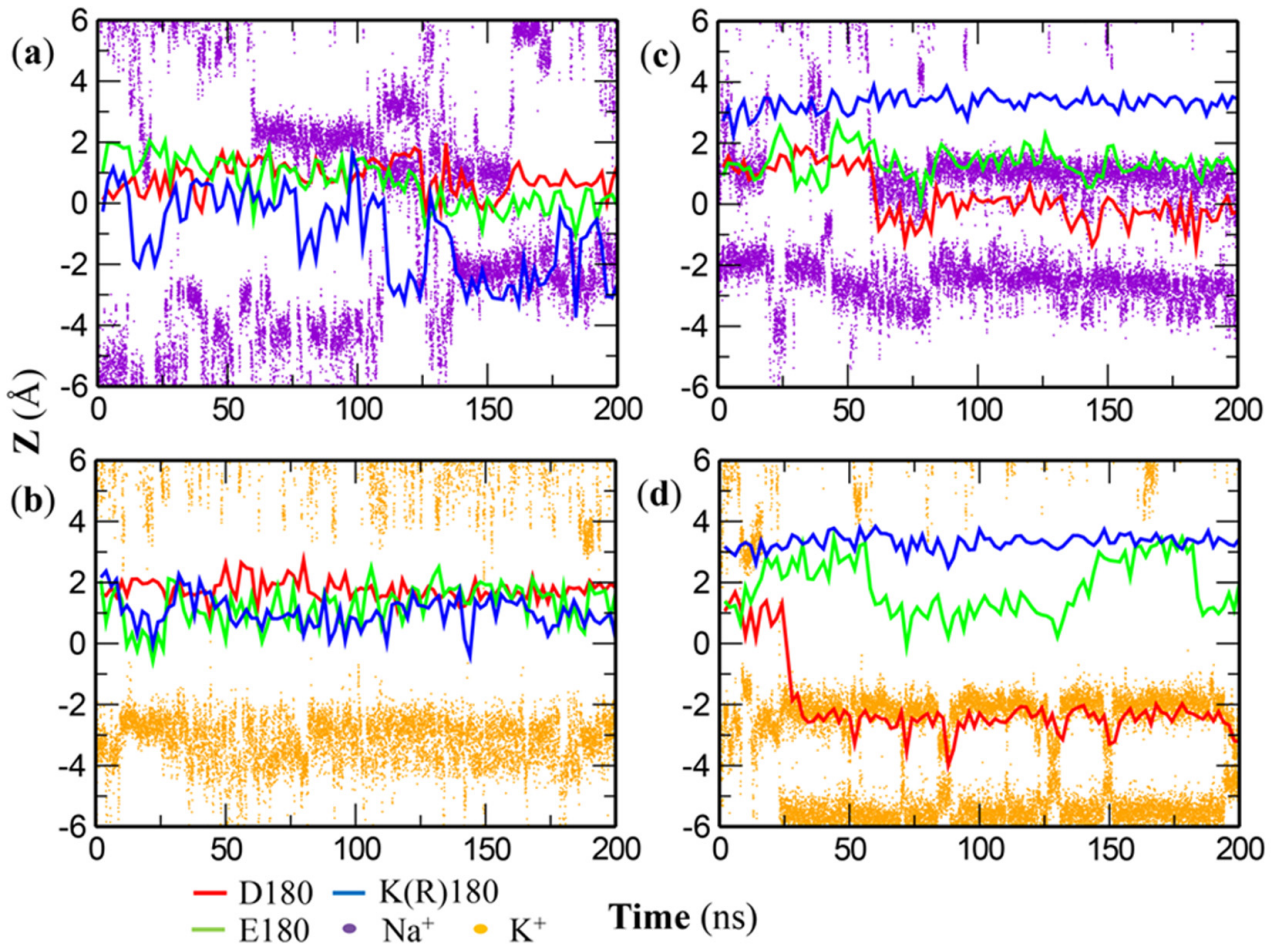
Time-dependent ion occupancy in the SF of the DEKA (a, b) and DERA (c, d) mutants of Na_v_Rh for Na^+^ (a, c) and K^+^ (b, d) ions in the a-system (IDs 13–16 in Table B in S1 File). The vertical axis is the relative distance of the cation to the geometric center of the SF along the z-axis. Violet and orange dots are used to represent the vertical positions of Na^+^ and K^+^ ions respectively. The red and green lines reflect the center of carboxylate oxygen atoms of Asp180 and Glu180 respectively. The blue line describes the position of the side-chain ζ-nitrogen (NZ) atom of Lys180 (**a**, **b**) or the ζ-carbon (CZ) atom of Arg180 (**b**, **d**).

**Fig 5:**
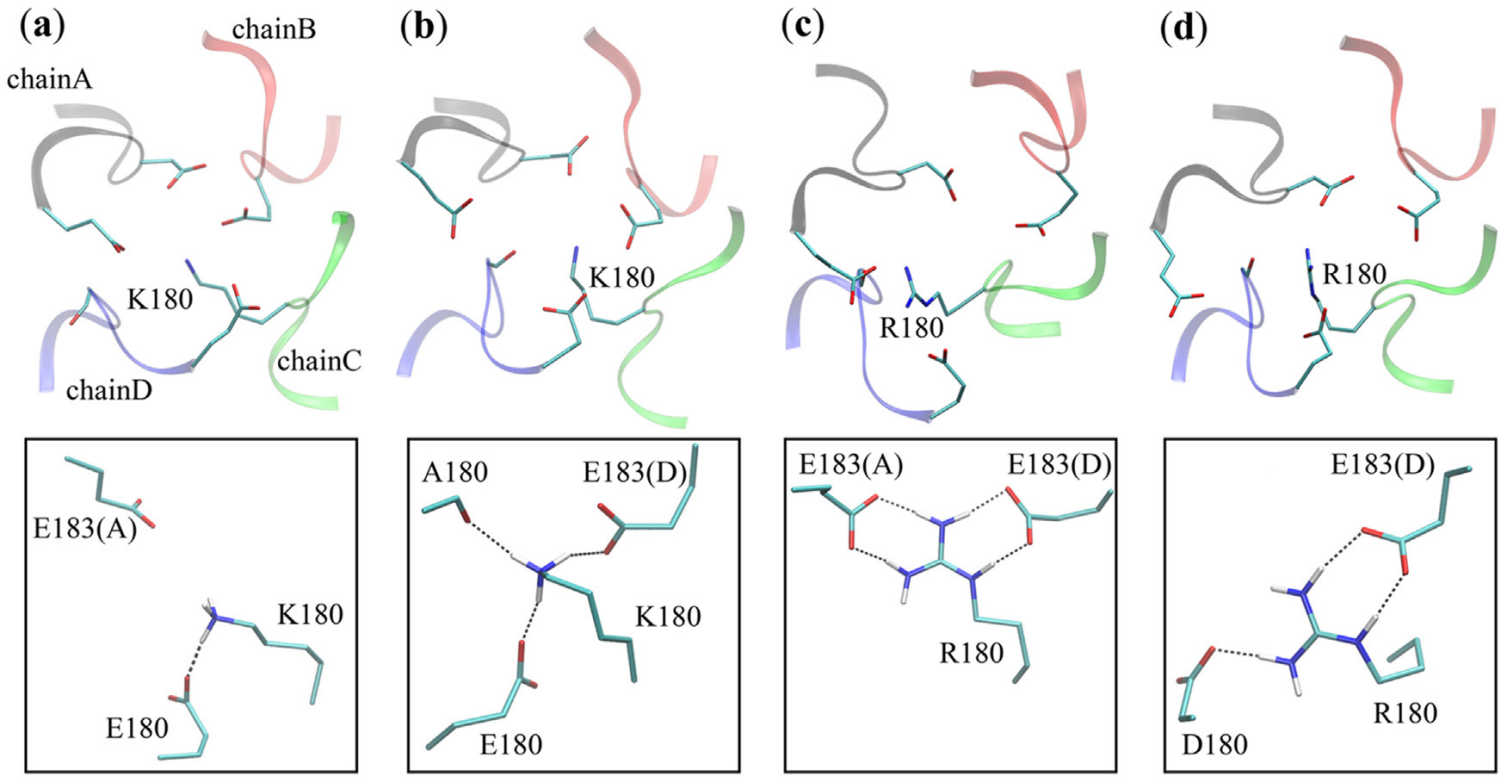
Structural comparison on the hydrogen bonding interactions between the side chains of Lys180/Arg180 and other residues. **(a)** For the DEKA mutant in the **a**-system (ID 13 in Table B in S1 File), Lys180 side chain can form only one stable hydrogen bond with Glu180. **(b)** For the DEKA mutant in the **d**-system (ID 17 in Table B in S1 File), Lys180 side chain can form concurrent hydrogen bonds with the carboxylate groups of Glu180 and Glu183 in chain D as well as the backbone carbonyl oxygen atom of Ala180. **(c)** For the DERA mutant in the **a**-system (ID 15 in Table B in S1 File), Arg180 side chain can form concurrent hydrogen bonds with the carboxylate groups of Glu183 residues in both chain A and chain D. **(d)** For the DERA mutant in the **d**-system (ID 19 in Table B in S1 File), Arg180 side chain can form concurrent hydrogen bonds with the carboxylate groups of Asp180 and Glu183 in chain D.

In the simulations of the **a**-system (IDs 13–16 in Table B in S1 File), the first cation can quickly enter the SF and favorably binds at the inner binding site (Site_INT_) that is located between Site_CEN_ and Site_IN_ (Figs 3 and 4). For both the DEKA and DERA mutants in NaCl (IDs 13&15 in Table B in S1 File), the second Na^+^ ion can stably bind at an outer binding site overlapping with Site_HFS_ (Fig 3a and 3c), where it favorably interacts with the carboxylate groups of Asp180 and Glu180, as indicated by the co-localization of ion and residue side chains in the z-axis (Fig 4a and 4c). In contrast, simulations on the DEKA and DERA mutants in KCl show quite different binding behaviors of the second K^+^ ion. In the DEKA mutant (ID 14 in Table B in S1 File), the second K^+^ ion occasionally reaches the DEKA ring but can rarely stay at the place (Figs 3b and 4b; Table D in S1 File). In the DERA mutant (ID 16 in Table B in S1 File), however, the second K^+^ ion favorably interacts with the side chain of Asp180, after the downward shift of the latter by >2 Å in the z-axis at 25 ns (Figs 3d and 4d). Because of the structural change in chain A (possibly due to the Arg180-Glu183(A) interaction, see Fig 5 and the following section for details), the outer K^+^ binding site in the DERA mutant apparently fall in the scope of Site_CEN_ (Fig 3d). Therefore, the traditional naming protocol of ion binding sites based on the static Na_v_Ab crystal structure is inadequate to describe the interactions between cations and the mammalian Na_v_ channels, due to the structural variation of proteins. Considering that cations in the outer binding site can form favorable interactions with the carboxylate groups of Asp180 and/or Glu180, we denoted this site as the outer constriction site (Site_OC_, pointed by arrows in Fig 3), where “outer” is used to distinguish it from the constriction region at the cytosolic entrance of PD. In summary, the DEKA mutant shows significantly different binding propensities for Na^+^ and K^+^ ions at the Site_OC_, while such distinction diminishes in the DERA mutant. These observations therefore agree with the mutational experiments.

In the simulations of the **d**-system (IDs 17–20 in Table B in S1 File), the first cation can also quickly approach Site_INT_ (Figs F and G in S1 File), although this site for K^+^ ion spans over the scopes of Site_CEN_ and Site_IN_ in the DERA mutant (Figs F and G in S1 File, panel d). Similar to the **a**-system, the second Na^+^ ion can stably bind at Site_OC_ in both DEKA and DERA mutants (Figs F and G in S1 File, panel ac; Table D in S1 File). Although the second K^+^ ion approaches Site_OC_ more frequently in DERA mutant than in DEKA mutant (Figs F and G in S1 File, panel bd; Table D in S1 File), its occupancy at Site_OC_ in the DERA mutant is substantially weaker than in the **a**-system (Table D in S1 File). Notably, the vertical shift of Asp180 side chain was not observed in the **d**-system, and Site_OC_ therefore overlaps with Site_HFS_ well.

In conclusion, the significant difference between the binding propensities of Na^+^ and K^+^ ions at Site_OC_ in the DEKA mutant vanishes completely in the **a**-system DERA mutant and becomes moderately reduced in the **d**-system DERA mutant, indicating that the K-to-R mutation changes the Na^+^/K^+^ selectivity by affecting the cation binding propensity at Site_OC_. Consequently, the following research will focus on the cation binding behavior at this site.

### Analysis of ion binding at Site_OC_ and interaction partners for Lys/Arg

We first analyzed the cation binding patterns at Site_OC_ for all simulations in both **a**-system and **d**-system. In addition, since our previous work suggested strong correlation between the electrostatic repulsion of Lys and Na^+^/K^+^ selectivity, we also checked the interaction partners for Lys/Arg side chains and their effects on the relative positions of these positively charged groups within the SF pore.

In the **a**-system, projections of the cation as well as the DEKA/DERA side chains in the xy-plane suggest that cation at Site_OC_ frequently interacts favorably with the carboxylate groups of Asp180 and Glu180, although the rare sampling and the sparse distribution of K^+^ ion in the DEKA mutant indicate extremely weak binding (Fig H in S1 File, panel a-d). In the DEKA mutant, structural flexibility of Lys180 side chain allows its terminal amino group to overcome its pre-formed salt bridge with Glu183 of chain A and to traverse relatively freely within the pore (see the scattered distributions in the Z-R space in panel ab of Fig I in S1 File). Despite the large freedom of movement, the Lys180 side chain tends to form favorable hydrogen bonds (in ~50% of the frames, see IDs 13&14 in Table E in S1 File) with the opposite Glu180, and sometimes forms an extra hydrogen bond with Glu183 in chain D (Fig 5a and panel ab of Fig J in S1 File). Such an interaction pattern helps position the amino group of Lys180 close to the center of SF pore (see the relatively small R values in panel ab of Fig I in S1 File), a posture in which Lys180 can effectively impose electrostatic repulsion on the bound cation. It is worth mentioning that constitutive (100% of simulation time) hydrogen bonding interaction between Lys side chain and one specific residue (e.g., Ser in the work by Mahdavi *et al*. [44]) was not observed in any of our simulations. In the DERA mutant, the guanidine group of Arg180 side chain remains close to its initial positions (see the localized distributions in the Z-R space in panel cd of Fig I in S1 File), where it forms stable bifurcate hydrogen bonds (in >67% of the frames, see IDs 15&16 in Table E in S1 File) with Glu183 residues of both chain A and chain D (Fig 5c and panel cd of Fig J in S1 File). Consequently, the Arg side chain tends to line along the side wall of SF pore (see the relatively large and localized R values in panel cd of Fig I in S1 File), which therefore greatly weakens the electrostatic repulsion imposed on the bound cations.

In the **d**-system, although the projections of Na^+^ ions show similar patterns to the **a**-system, the sparse distributions of K^+^ projection points negate the good coordination (Fig H in S1 File, panel e-h). Thus, K^+^ ions are less favorably bound at Site_OC_ of the DERA mutant in the **d**-system than in the **a**-system. In the DEKA mutant, the movement of Lys180 side chain is similar to the **a**-system, with the terminal amino group relatively freely traversing in the SF pore (Fig I in S1 File, panel ef). Besides the stable hydrogen bonds with the opposite Glu180 (in ~50% of the frames, see IDs 17&18 in Table E in S1 File), Lys180 side chain sometimes forms additional partial hydrogen bonds with Ala180 and Glu183 in chain D (Fig 5b and panel ef of Fig J in S1 File). Consequently, despite the substantial freedom in movement, the amino group of Lys180 is inclined to stay near the center of SF pore. In the DERA mutant, although Arg180 side chain can form stable bifurcate hydrogen bonds (in >80% of the frames, see IDs 19&20 in Table E in S1 File) with Glu183 of chain D (Fig 5d and panel gh of Fig J in S1 File), its close interaction with Glu183 of chain A as found in the **a**-system is not observed here. In addition, Arg180 side chain can form a single hydrogen bond with the opposing Asp180 (Fig 5d and panel gh of Fig J in S1 File), which thus pulls the Arg180 side chain to swing slightly in the inward direction as compared to the **a**-system (compare panel gh and panel ef of Fig I in S1 File). Nevertheless, the bifurcate attraction from Glu183 of chain D still guarantees the guanidine group of Arg180 to deviate from the center of SF pore, thereby partially weakening the electrostatic repulsion.

In summary, the close interaction with Glu180 facilitates positioning the amino group of Lys180 at the center of the SF pore, while the bifurcate interactions with Glu183 residue in chain D and possibly with the one in chain A could attract the Arg180 side chain towards the side wall of SF pore. We hypothesize that strong electrostatic repulsion on the bound cation is essential for sustaining the strong Na^+^/K^+^ selectivity at Site_OC_, and that the Arg side chain in the DERA mutant tends to be attracted away from the center of the SF pore by the bifurcate interactions with acidic residues in the outer ring, which eventually impairs the repulsive power and thus the Na^+^/K^+^ selectivity. This hypothesis will be quantitatively validated by free energy calculations in the following section.

### Na^+^/K^+^ selectivities for the main cation binding modes at Site_OC_

To validate our hypothesis, we need to quantitatively evaluate the Na^+^/K^+^ selectivity at Site_OC_. Visual inspection on the ion binding patterns indicates not only great inter-system differences (e.g., Na^+^ vs. K^+^ and DEKA vs. DERA) but also substantial structural variations within each single system. Therefore, for each system, we first classified the cation binding modes at Site_OC_ through structural clustering based on the relative positions of the bound cation and the side-chain oxygen/nitrogen atoms of DEKA/DERA residues, and then adopted the free energy perturbation (FEP) method [57, 58] to calculate the relative Na^+^/K^+^ binding affinity (*ΔΔG(Na*^*+*^*→K*^*+*^*)*) for the main cation binding modes by restraining the ion position in each mode using a flat-bottom potential (see the detailed simulation protocol in the S1 File). In this way, the negative effect of structural variation on the convergence of FEP calculation can be effectively eliminated. Three largest clusters for each system were extracted from the last 150 ns of the equilibrium simulations (Table F in S1 File). Considering the generally low occupancy of third clusters, only the first two clusters were considered as the main cation binding modes. In addition, because FEP calculation was hard to approach convergence for tiny clusters, for the simulations with extremely weak cation binding (e.g., the K^+^ binding in the DEKA mutant), only the top binding mode was taken for free energy evaluation.

In the **a**-system, except for the limited presence of K^+^ at Site_OC_ in the DEKA mutant, the cations are densely populated between the carboxylate groups of Asp180 and Glu180 in all main binding modes. In addition, different from Lys180, the Arg180 side chain is typically attracted outwards, possibly by the bifurcate interactions with Glu183 residues in the outer ring (Fig I in S1 File and Table E in S1 File). Moreover, free energy calculations suggest that all main cation binding modes in the DEKA mutant are Na^+^-selective, with *ΔΔG(Na*^*+*^*→K*^*+*^*)* > 1.1 kcal/mol (Table 1). In contrast, the DERA mutant is strongly Na^+^-selective (*ΔΔG(Na*^*+*^*→K*^*+*^*)* > 3 kcal/mol) for the main binding modes of Na^+^ ions, but becomes neutral (*ΔΔG(Na*^*+*^*→K*^*+*^*)* ≈ 0) or weakly K^+^-selective (*ΔΔG(Na*^*+*^*→K*^*+*^*)* < 0) for the main binding modes of K^+^ ions (Table 1).

**Table 1:** The relative binding affinities at Site_OC_ for the main cation binding modes in the DEKA and DERA mutants.

ID	System	Main binding modes for ions	*ΔΔG(Na*^*+*^*→K*^*+*^*)* (kcal/mol)	
			Mode #1	Mode #2
13	DEKA-**a**	Na^+^	2.56±0.40	1.17±0.25
14		K^+^	1.57±0.34	NA
15	DERA-**a**	Na^+^	3.84±0.60	3.66±0.22
16		K^+^	-0.46±0.43	0.09±0.83
17	DEKA-**d**	Na^+^	2.84±0.31	3.80±0.18
18		K^+^	3.45±0.63	NA
19	DERA-**d**	Na^+^	3.35±0.21	3.94±0.22
20		K^+^	2.39±0.18	2.32±0.17

In each case, the calculation was repeated for three times to estimate the mean and standard deviation. The original data for this table are listed in Table J in S1 File.

In the **d**-system, despite the weak binding of K^+^ ions, both cations are located near the center of the carboxylate groups of Asp180 and Glu180. The guanidine group of Arg180 side chain swing outwards to a less extent than in the **a**-system (Fig I in S1 File). According to the free energy evaluations, the DEKA mutant strongly prefers Na^+^ ions at Site_OC_, with *ΔΔG(Na*^*+*^*→K*^*+*^*)* > 2 kcal/mol for all main binding modes (Table 1). Despite the similarly strong Na^+^-preference (*ΔΔG(Na*^*+*^*→K*^*+*^*)* > 2 kcal/mol) in the DERA mutant, the Na^+^/K^+^ selectivities in the main binding modes of K^+^ ions are slightly weakened (by ~1 kcal/mol), when compared to the main binding modes of Na^+^ ions (Table 1).

Therefore, most cation binding modes at Site_OC_ are Na^+^-selective, while a few modes are neutral or slightly K^+^-selective. In order to understand why these binding modes exhibit such a wide range of Na^+^/K^+^ selectivity, we calculated the number of coordinating carboxylate oxygen atoms for each main binding modes. According to the QM/MM calculations by Azam *et al*., the coordinating distance between Na^+^/K^+^ and oxygen atoms falls in the range of 2.39~2.99 Å and 2.80~3.65 Å, respectively [59]. Here, we used the means of the lower and upper limits as the cutoff values (2.69 Å and 3.22 Å for Na^+^ and K^+^ ions respectively) to differentiate coordinating and non-coordinating oxygen atoms (Table G in S1 File). In all Na^+^-selective binding modes, the cation is well coordinated by at least three carboxylate oxygen atoms, a geometry that suppresses the appropriation of other oxygen atoms (e.g., from waters) and therefore intrinsically favors small cations with a low coordination number (the maximal coordination numbers for Na^+^ and K^+^ ions are 6 and 8 respectively). As a contrast, in the neutral or slightly K^+^-selective binding modes (e.g., the K^+^-binding modes of the DERA mutant in the **a**-system), the cation is coordinated by no more than two carboxylate oxygen atoms. Notably, when interacting with proteins, cations can be coordinated by other oxygen atoms from proteins, including the backbone carbonyl oxygen and side-chain hydroxyl oxygen. However, none of these candidate oxygen atoms are ion-coordinating in the neutral or slightly K^+^-selective binding modes (Table H in S1 File). Therefore, in these binding modes, cations are only loosely surrounded by protein oxygen atoms, an environment that allows the appropriation of more water oxygen atoms to fulfill the maximal coordination number of K^+^ ions.

Although both Lys and Arg carry one unit of positive charge, they have different repulsive powers on cations. In a control experiment, we simulated the interaction between a Lys/Arg (chemically blocked at both ends) and one Na^+^/K^+^ ion in a cubic water box, and eventually obtained the dependence of electrostatic repulsion on the distance between cation and the amino/guanidine group. As shown in Fig K in S1 File, the repulsion weakens with rise of interacting distance. However, at short distance (< 5 Å), Lys is much more repulsive to Na^+^/K^+^ ions than Arg. Subsequently, we calculated the distances between cations and the terminal nitrogen atoms in the side chains of Lys180/Arg180 for all main cation binding modes. As shown in Table G in S1 File, for most binding modes of the DEKA mutant, the amino group of Lys is close to the bound cation (< 5 Å), imposing strong electrostatic repulsion. The only exception occurs in the 2^nd^ Na^+^-binding mode of the DEKA mutant in the **a**-system, possibly because of the substantial structural flexibility of the Lys side chain. In this binding mode, the cation has more freedom to adjust its position (see its large distance to OE2 of Glu180 in Table G in S1 File), which slightly weakens the Na^+^-preference. In contrast, the guanidine group of Arg frequently stays away from the bound cation. For instance, the guanidine-cation distance in the K^+^ binding modes is usually greater than 5 Å. Especially noteworthy, in the **a**-system where the Arg side chain forms bifurcate hydrogen bonds with the carboxylate groups of two Glu residues in the outer ring (Fig 5c), the guanidine-cation distance exceeds 5.5 Å in all cation binding modes, which thus allows the emergence of neutral or slightly K^+^-selective binding modes, since under the greatly weakened electrostatic repulsion, the cation can partially leave the carboxylate oxygen atoms of Asp180 and Glu180 for better water coordination.

**Fig 6:**
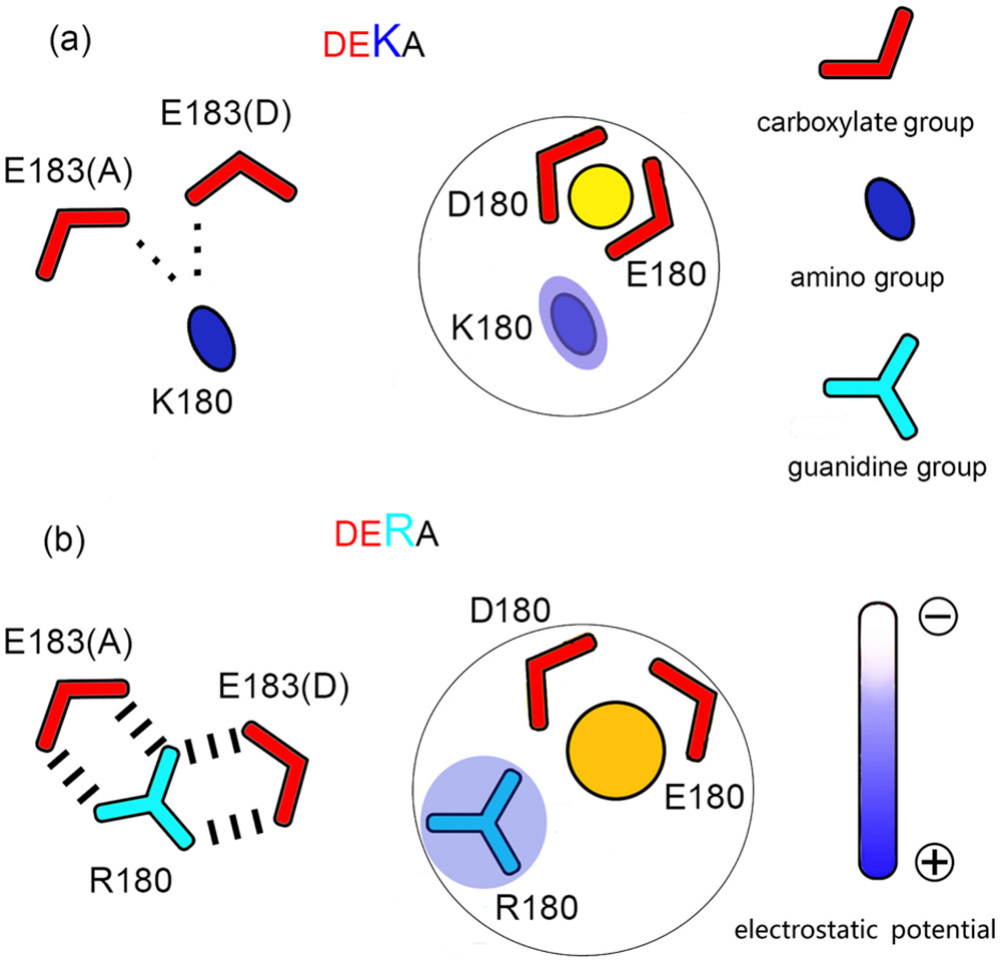
Diagram of the model to explain the difference in ion selectivity between the DEKA and DERA mutants. Na^+^ and K^+^ ions are represented by yellow and orange spheres respectively. **(a)** In the DEKA mutant, Lys180 side chain tends to protrude into the center of the SF, repelling the cation to bind at the Na^+^-preferred sub-location sandwiched by at least three carboxylate oxygen atoms of Asp180 and Glu180. **(b)** In the DERA mutant, because of the stable bifurcate interactions with Glu183 residues (in chain A and D), Arg180 side chain tends to line along the side wall of the SF pore. This conformational difference as well as the lower charge density on the guanidine group jointly weaken the electrostatic repulsion and allow the cation to sample the less Na^+^-selective or even K^+^-preferred sub-locations coordinated by no more than two carboxylate oxygen atoms of Asp180 and Glu180.

Combining all of the results, we can propose a molecular model for the Na^+^/K^+^ selection in the DEKA and DERA mutants of Na_v_Rh (Fig 6). The difference in Na^+^/K^+^ selectivity primarily arises from distinct cation binding behaviors at Site_OC_, the binding site where the DEKA/DERA residues are located. Albeit structurally flexible, the Lys180 side chain in the DEKA mutant tends to protrude towards the center of SF pore, forming favorable interaction with the carboxylate group of the opposite Glu180. In this posture, Lys180 imposes strong electrostatic repulsion on the cation bound at Site_OC_, driving it to stay between the carboxylate groups of Asp180 and Glu180 and to adopt a Na^+^-preferred binding mode by forming close coordination with at least three carboxylate oxygen atoms. In the DERA mutant, Arg180 side chain is attracted away from the center of SF pore by the bifurcate interactions with Glu183 residues in the outer ring, which substantially weakens the electrostatic repulsion to the cation. Therefore, in addition to the Na^+^-preferred binding mode, the cation is allowed to adopt the neutral or K^+^-preferred binding mode, by partially leaving the center of the carboxylate groups of Asp180 and Glu180 and thus forming coordination with no more than 2 carboxylate oxygen atoms.

### Structural difference between the side chains of Lys and Arg

According to our molecular model, the disruption of Na^+^/K^+^ selectivity in the DERA mutant primarily arises from stable interactions between the Arg180 side chain and the carboxylate groups of Glu183 residues in the outer ring (Fig 5c and Table E in S1 File). Steady interactions between Lys180 and Glu183 residues, however, were not observed in the DEKA mutant, although the constraints applied in the pre-equilibrations guarantee such interactions at the beginning of equilibrium simulations (Fig 5 and Fig J in S1 File; Table E in S1 File). To further validate our model, we introduced additional mutations to Glu183 of chain D and then simulated the interaction between this residue and Arg180. In the first set of control simulations (IDs 21&22 in Table B in S1 File), the Glu183 in chain D was mutated to Asp, a residue appearing at the corresponding position in mammalian Na_v_ channels (see Table A in S1 File). For this mutant, the distances and hydrogen bonds between the Arg180 side chain and the carboxylate groups of Asp183 in chain D as well as Glu183 in chain A are generally unperturbed (Fig L in S1 File, panel a; IDs 21&22 in Table E in S1 File), and the Arg180 side chain keeps staying away from the center of SF pore (Fig M in S1 File, panel a). In the second set of control simulations (IDs 23&24 in Table B in S1 File), Glu183 in chain D was mutated to Ala to disrupt the bifurcate interaction. In this mutant, the hydrogen bonds between Arg180 and Glu183 in chain A are completely broken (Fig L in S1 File, panel b; IDs 23&24 in Table E in S1 File). Moreover, the Arg180 side chain swings inwards to form hydrogen bonds with residues at the constriction site (Fig M in S1 File, panel b; IDs 23&24 in Table E in S1 File), thereby positioning its terminal guanidine group to the center of SF pore and partially closing the channel (Fig M in S1 File, panel b). These two sets of control simulations confirm our proposition that the positional preference of the Lys and Arg side chains is actually determined by their propensity to form steady interactions with the acidic residues in the outer ring.

*Why can Arg form steady interaction with acidic residues*? Although the side chains of both Lys and Arg are positively charged, they show great distinction in the charge distribution. As shown by the electrostatic surface potentials calculated for both residues (Fig N in S1 File, top row), Lys has a denser local charge density on the smaller amino group, while the positive charge is more evenly distributed in the planar guanidine group of Arg. Residue topologies in the CHARMM36 force field also support this difference (Fig N in S1 File, middle row). The symmetric design of guanidine group thus allows the Arg side chain to form bifurcate hydrogen bonds with two carboxylate groups simultaneously. To test this hypothesis, we artificially changed the charge distribution in the Arg side chain to enhance the local charge density of one–NH_2_ (denoted as e* with the modified topology shown in the bottom row of panel b of Fig N in S1 File), and then simulated the structure in NaCl, starting from a conformation with the bifurcate interaction between Arg180 and Glu183 in chain A or chain D (IDs 25&26 in Table B in S1 File). The charge-redistributed Arg180 side chain quickly swings inwards and moves towards to the center of SF pore (Fig M in S1 File, panel cd), losing interaction with Glu183 in chain A/D (Fig L in S1 File, panel cd).

Apart from symmetry, the sparser charge distribution on the guanidine group weakens the electrostatic repulsion of the Arg side chain (Fig N in S1 File), thus partially impairing the Na^+^/K^+^ selectivity. In another control experiment, we artificially reduced the positive charge on the amino group of Lys180 in the DEKA mutant by 50% and 95% (called system 0.5e and 0.05e respectively with topologies shown in the bottom row of panel a of Fig N in S1 File), and then simulated the two modified structures in NaCl and KCl (IDs 27–30 in Table B in S1 File). For these two proteins, both Na^+^ and K^+^ ions can bind stably at Site_OC_ (Fig O in S1 File), indicating impaired Na^+^/K^+^ selectivity.

### Perspectives for the mammalian Na_v_ channels

In the equilibrium simulations, the Arg180 side chain in the DERA mutant mainly exhibits two classes of interaction patterns (Fig 5c and 5d), which either completely or partially impair the Na^+^/K^+^ selectivity at SiteOC. In the major class of interaction pattern, the Arg sidechain is drawn away from the center of SF pore by its stable interaction with the two acidic residues in both the neighboring and the opposite chains. Considering the conservation of these two acidic residues at the outer ring of the SF in all mammalian Na_v_ channels (Table A in S1 File), the interaction pattern proposed here may be conserved in mammalian Na_v_ channels, which may weaken the electrostatic repulsion of Arg and disrupt the Na^+^/K^+^ selectivity after the K-to-R mutation. The loss of ion selectivity in the mammalian DERA mutant may arise from the joint contributions of two intrinsic features of the Arg side chain: the symmetric planar structure and the sparse charge density of guanidine group. The former stabilizes the Arg side chain in a specific interaction pattern (with two acidic residues concurrently) to inhibit its protrusion towards the center of SF pore, while the latter directly weakens the electrostatic repulsion exerted on the cations.

According to our previous work [45] and the present study, the sub-location sandwiched by the carboxylate groups of Asp and Glu at the constriction site is highly Na^+^-selective. The Na^+^/K^+^ selectivity of the constriction site is facilitated by the presence of a positively charged residue, which drives the permeating cation to penetrate through this highly Na^+^-selective sub-location by electrostatic repulsion. However, the Na^+^/K^+^ selectivity will be impaired either if its side chain has limited length and/or flexibility to impose the repulsion or if the positive charge is sparsely distributed on the side chain, since the repelling power of this residue is greatly undermined in both cases. In other words, the proper ion selectivity in mammalian Na_v_ channels is sustained under of help of a densely positively charged residue with flexible side chain at the constriction site. Among the 20 kinds of natural amino acids, Lys is the only candidate that fulfills both requirements. Consequently, Lys is completely conserved in all mammalian Na_v_ channels.

## Supporting Information

S1 File Including the detailed simulation protocol, Figs A-Q and Tables A-J. (PDF)
