# Supplementary material for: Lysine and the Na+/K+ Selectivity in Mammalian Voltage-Gated Sodium Channels
Source: PLoS One. 2016 Sep 1;11(9):e0162413. doi: 10.1371/journal.pone.0162413 (PMC5008630; doi:10.1371/journal.pone.0162413)
Supplement: S1 File — (PDF) [file pone.0162413.s001.pdf]

Detailed simulation protocol

Common parameters of all equilibrium simulations

The structural model was generated in a similar way to our previous work [1]: the Na_vRh channel (PDB code: 4DXW) crystal structure was taken as the initial model; after removing the voltage-sensing domain (VSD), the pore domain (PD) with residues 118-227 in all chains was inserted into the POPC bilayer (containing ~187 POPC molecules) for simulation. The mutant protein structures were generated using the "Mutator" plugin of VMD 1.9.1 [2]. The system was then immersed in TIP3P water (~16000 water molecules) and electrically neutralized by 150 mM XCl, where X corresponds to Na^+ or K^+. All simulations were conducted in an NPT ensemble using NAMD 2.9 [3], where the pressure and temperature were held at 1 atm and 310 K by the Nose-Hoover Langevin piston [4, 5] and the Langevin thermostat [5] respectively. The CHARMM36 force field [6, 7] was utilized to simulate the system with CMAP correction [8] and NBFIX [9]. The van der Waals interaction was cutoff at 12 Å using a smooth switch at 10 Å. The electrostatic energy was estimated using the Particle Mesh Ewald (PME) method [10] with periodic boundary conditions (PBC) engaged. The SETTLE algorithm [11] was used to restrain all hydrogen atoms whenever the time step was chosen as 2 fs.

The unbiased systems (IDs 1-8 in Table B) followed 7 steps of pre-equilibration (Fig C, panel a-c). In step 1, the systems were first energy-minimized for 2000 steps, and were then equilibrated using a time step of 1 fs for 0.5 ns, with all atoms fixed except the lipid tails, in order to relax the lipid-protein interaction. In steps 2-6, in order to relax the protein-water interaction, these systems were equilibrated for 1 ns at every step, after 2000 steps of minimization at the beginning of step 2. The positional restraints were applied in the following manner: Step 2, 1 kcal/mol on all atoms of the protein; Step 3, 1 kcal/mol on all non-hydrogen atoms of the protein; Step 4, 1 kcal/mol on the backbone atoms of the protein; Step 5, 0.1 kcal/mol on all α-carbon atoms of the protein; Step 6, 0.01 kcal/mol on all α-carbon atoms of the protein. In addition, in steps 2-6, all ions were restrained at least 10 Å away from the SF using a

flat-bottom potential of 1 kcal/mol. In step 7, all systems were further relaxed for about 10 ns with a positional restraint of 0.01 kcal/mol applied on the α-carbon atoms of the N-terminal S4-5 helices (residues 118-123). After the above pre-equilibration steps, the equilibrium simulations were conducted with the same restraint as in step 7 of the pre-equilibration (a positional restraint of 0.01 kcal/mol applied on α-carbon atoms of the N-terminal S4-5 helices) to eliminate the negative influence of removing VSD from the protein (Fig C, panel d). The time step was changed to 2fs in steps 2-7 in the pre-equilibrations as well as in the equilibrium simulations.

Constraint to restrict the distance between Lys/Arg and Glu in the outer ring

In our previous work [1], a flat-bottom constraint was applied to restrict the distance between the ζ-nitrogen (NZ) atom of Lys180 and the carboxylate carbon (CD) atoms of Glu183 residue in chain D. Here, to test the interaction partner of Lys180 (IDs 9-12 in Table B), an additional constraint was applied in the pre-equilibration stage to maintain the interaction between Lys180 and Glu183 in chain A, B, C and D respectively (Fig C, panel e). Specifically, a flat-bottom potential was applied to restrain the distance between the NZ atom of Lys180 and the δ-carbon (CD) atom of Glu183 within a cutoff. In steps 2-7, the force constant of the potential was gradually reduced from 5 kcal/mol to 1 kcal/mol, and the distance cutoff was raised from 4 Å to 6 Å (Steps 2-6: 5 kcal/mol and 4 Å; Step 7: 1 kcal/mol and 6 Å). The pre-equilibration in step 7 was extended to 20ns to fully relax the systems. This constraint on the distance was completely removed in the equilibrium simulation.

In the following simulations on the DEKA mutant (IDs 13, 14, 17 & 18 in Table B), the constraint was applied in the same protocol as described above, but only on the Glu183 residues in chain A and chain D (denoted as **a**-system and **d**-system respectively). In the simulations on the DERA mutant (IDs 15, 16, 19 & 20 in Table B), a similar constraint was applied to restrain the distance between the ζ-carbon (CZ) atom of Arg180 and the CD atom of Glu183 in chain A and chain D. This constraint

on distance was completely removed in the following equilibrium simulations on both DEKA and DERA mutants. According to the root-mean-square-distance (RMSD) analysis, all systems are stable in the equilibrium simulations (for example, RMSD profiles of IDs 13-20 in Table B are shown in Fig P).

Initial structures and simulation parameters for the control simulations

Numerous simulations (IDs 21-30 in Table B) were performed as controls to test our molecular model. The initial structures of these simulations were taken from centroid of the top structural cluster in corresponding equilibrium simulation (see details in Table B), and were then simulated for 50 ns after 1 ns pre-equilibration where all heavy atoms were constrained with a force constant of 0.01 kcal/mol. In the equilibrium simulations, only the weak positional restraint of 0.01 kcal/mol on the α-carbon atoms of the N-terminal S4-5 helices was retained to avoid the negative influence of removing VSD from the protein. In order to evaluate the effect of charge density on the cation selection, the partial charges on the side chains of Lys and Arg were modified (IDs 25-30 in Table B), as shown in Fig N.

Electrostatic repulsion between Lys/Arg and Na^+/K^+ ions

To compare the repulsive interactions between Lys/Arg and Na^+/K^+ ions, we built 4 cubic water boxes with side length of 30 Å and placed one cation (Na^+/K^+ ion) and one amino acid (Lys/Arg) into each box. The N- and C-termini of the Lys/Arg residue were blocked by ACE and NME groups respectively. During the simulation, the cations are fully free, while the backbone atoms of Lys/Arg are restrained by a positional restraint of 1 kcal/mol. After 10 ns of equilibrium simulation, the minimal distance (N_{MIN}) between cation and any side-chain nitrogen atoms of Lys/Arg was evaluated. The electrostatic energy between cation and Lys/Arg was calculated using the “NAMD Energy” plugin of VMD 1.9.1. To generate the dependence curve of

electrostatic energy upon distance, cation-residue pairs located within a bin size of 0.5 Å were grouped together and their mean was taken as the energy value at the distance centered at the corresponding bin.

Structural analysis on the trajectories from equilibrium simulations

To obtain the main cation binding modes, the structural snapshots from the last 150 ns trajectories of the equilibrium simulations (IDs 13-20 in Table B) were clustered based on the relative positions of the following atoms: the cation (Na^+ or K^+) at $Site_{OC}$, ε-oxygen (OE1 and OE2) atoms of Glu180, δ-oxygen (OD1 and OD2) atoms of Asp180, η-nitrogen (NZ) atom of Lys180 or ε/η-nitrogen (NE, NH1 and NH2) atoms of Arg180, as well as carbonyl oxygen atom (O) of Ala180. Before clustering, all PD structures were aligned against the crystal structure. A cutoff of 0.65 Å was used to produce large clusters.

The hydrogen bonds were evaluated using the "Hydrogen Bonds" plugin of VMD 1.9.1, using 3 Å and 20° as the cutoff values for the donor-acceptor distance and the donor-hydrogen-acceptor angle respectively.

The free energy perturbation (FEP) simulations

The relative binding affinities ($\Delta\Delta G(Na^+ \rightarrow K^+)$) for the main cation binding modes were evaluated by the FEP method [12, 13]. The initial structures of FEP simulations were obtained from the centroid of the top clusters. Time step was chosen as 1 fs while all other simulation parameters were identical to those in the equilibrium simulations. The same constraint was applied on the protein N-terminal S4-5 helices (residues 118-123) as in the equilibrium simulations. Moreover, the target cations were further restrained by a flat-bottom potential with a force constant of 10 kcal/mol/Å^2. Taking the average position of cations within each cluster as the reference, the target cation received a penalty when its deviation from the reference position exceeded a cutoff value, which was the radius of the spherical envelop

covering 80% data of the cluster.

After 2-3 ns pre-equilibrations, FEP simulations were conducted in both forward and backward directions for better accuracy, and the data were processed by the Bennett acceptance ratio (BAR) estimator [14, 15] using the "parse FEP" plugin of VMD 1.9.1. The dual-topology paradigm [16, 17] was adopted and the appearing Na^+/K^+ were forced to overlap with the vanishing K^+/Na^+ using a strong constraint (100 kcal/mol). The soft core potential [18] was used to avoid the "end-point catastrophes" and intra-molecular interactions were decoupled from the FEP calculations. The reaction coordinate was divided into 186 small windows. In each window, 30000-60000 steps of simulation were conducted to ensure convergence, which was judged by two criteria [15]: 1) nearly overlapping probability distributions of the energy changes in the forward and backward processes, $P(\Delta E_{forward})$ and $P(-\Delta E_{backward})$; and 2) small difference (< 2.5 kcal/mol) between the free energy values obtained from the two unidirectional calculations. The total simulation time of each FEP calculation was 15.2 ns and the calculation for each cation binding mode was repeated for three times to estimate the mean and standard deviation.

According to the thermodynamic cycle shown in Fig Q, $\Delta\Delta G(Na^+ \rightarrow K^+)$ can be derived using the following equation,

$$\begin{aligned}\Delta\Delta G(Na^+ \rightarrow K^+) &= \Delta G_{binding}(K^+) - \Delta G_{binding}(Na^+) \\ &= \Delta G_{site}(Na^+ \rightarrow K^+) - \Delta G_{bulk}(Na^+ \rightarrow K^+),\end{aligned} \quad (1)$$

where $\Delta G_{binding}(K^+)$ and $\Delta G_{binding}(Na^+)$ stand for the binding free energies of K^+ and Na^+ at the site respectively, while $\Delta G_{site}(Na^+ \rightarrow K^+)$ and $\Delta G_{bulk}(Na^+ \rightarrow K^+)$ represent the free energy changes arising in the $Na^+ \rightarrow K^+$ alchemical transformation at the binding site and in the bulk solution respectively. The protocol is intrinsically designed for evaluating the binding modes of Na^+ ions, since the bound Na^+ ion could be directly converted to K^+ ion through alchemical transformation to estimate $\Delta G_{site}(Na^+ \rightarrow K^+)$. For the binding modes of K^+ ions, the $\Delta G_{site}(Na^+ \rightarrow K^+)$ was derived from the negative value of the reverse alchemical transformation $\Delta G_{site}(K^+ \rightarrow Na^+)$.

The $\Delta G_{bulk}(Na^+ \rightarrow K^+)$ was evaluated in a control simulation as 18.47±0.05 kcal/mol (see Table I), which is very close to the experimental measurement of ~17

kcal/mol on the difference in hydration energies between Na^+ and K^+ ions [19, 20]. The control system was in the same dimension as the protein systems and was neutralized with 0.15 mol/L NaCl. A free Na^+ ion was mutated to K^+ ion to estimate the free energy. The simulation and FEP parameters were the same as the calculations for the cation binding modes, except that the simulation time was shortened to 6.64 ns because of the fast convergence.

Supplementary Figures

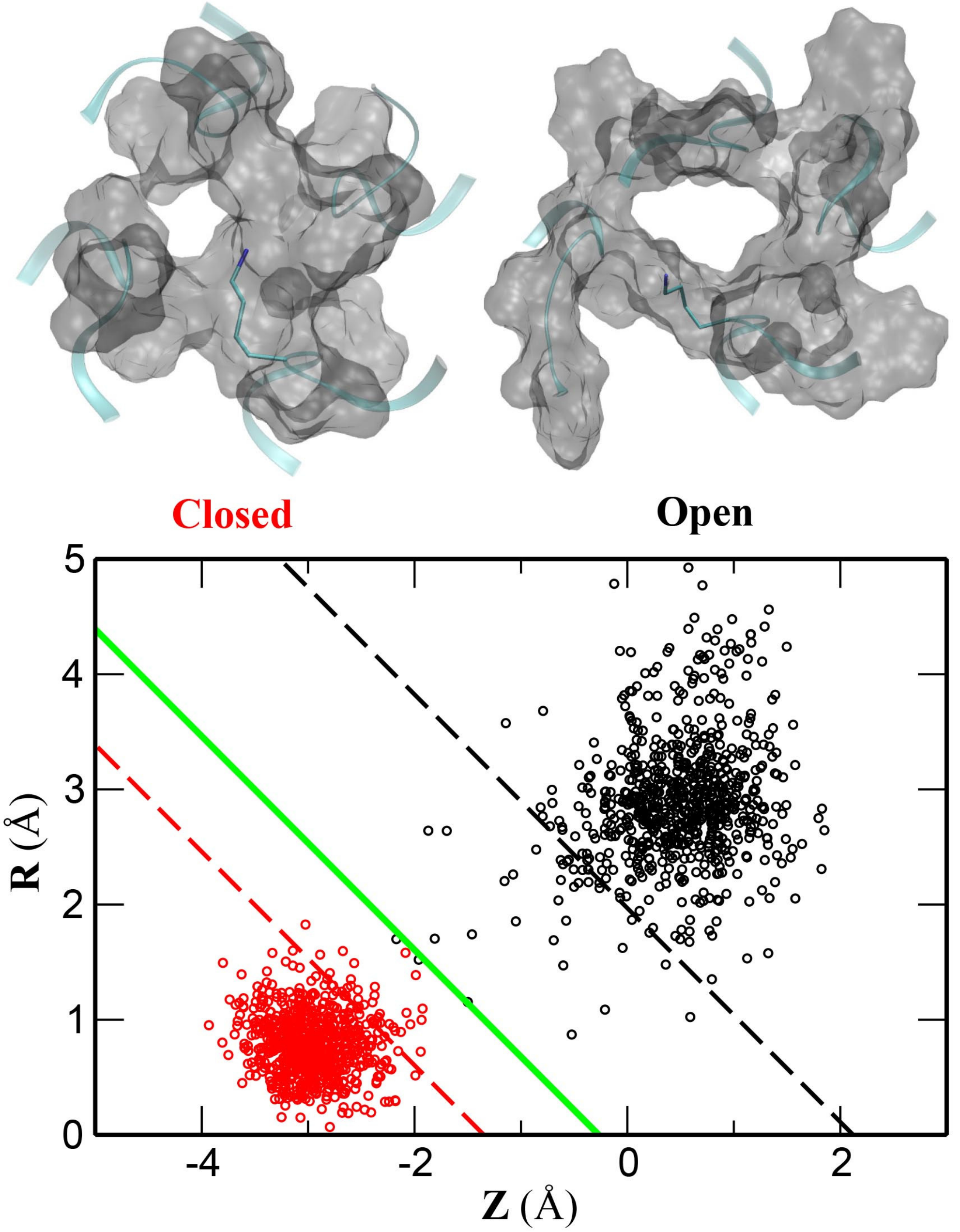

Fig A. Classification on the open and closed states for the DEKA mutant in two constraint-free short simulations (IDs 5-6 in Table B). Representative structures of the closed and open states for the SF are shown in the upper panel, where the surfaces of SF residues are shown as transparent, in the color of gray. In the lower panel, structures from the last 15 ns trajectories of both simulations are plotted in a 2D plane of R and Z, where Z stands for the position of the ζ-nitrogen (NZ) atom of Lys180

side chain along the z-axis and R represents the distance between NZ and the geometric center of the SF in the xy-plane that is parallel to the membrane bilayer. The closed and open structures are colored in red and black, respectively. The best linear separation between the two states (calculated using the SVM model) is colored in green. The dotted lines represent the linear boarders (parallel to the green line) for the regions covering 95% structural snapshots of the closed (red) and open (black) states, respectively.

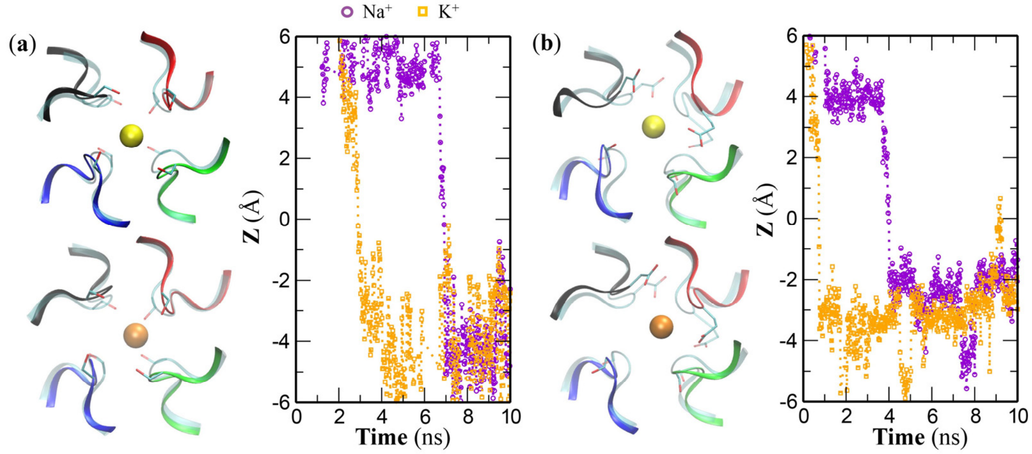

Fig B. The cation binding patterns in the SF of the WT (**a**) and DEAA mutant (**b**) of Na_vRh (IDs 1-4 in Table B). In each map, the left panel shows the representative structures extracted from the equilibrium simulations, where protein chains and ions are colored in the same scheme as in Fig 2. The right panel shows the time-series of the vertical positions of cations along the z-axis (Na^+ ions in violet vs. K^+ ions in orange).

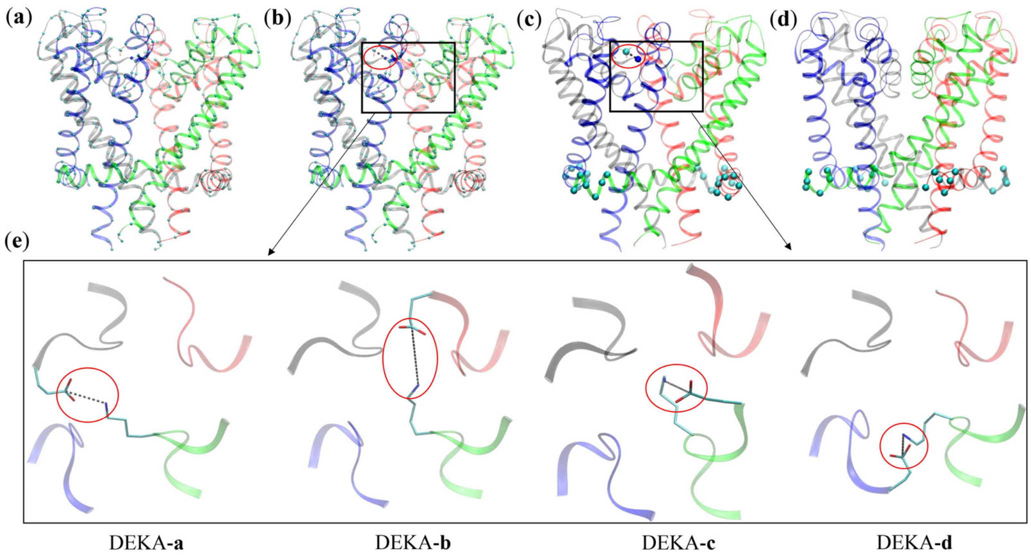

Fig C. The protocol for equilibrium simulations. Systems (IDs 1-20 in Table B) were pre-equilibrated in 7 steps (**a**-**c**). (**a**) Step 1, the protein was fixed while lipid tails and waters were allowed to move for 0.5 ns, after 2000 steps of minimization; (**b**) Steps 2-6, in order to relax the protein-water interaction, the positional constraints applied on the protein atoms (shown as tiny spheres) were relaxed gradually (from 1 to 0.01 kcal/mol) (see detailed information in the *Supplementary Methods*). (**c**) Step 7, all systems were further relaxed for 10/20 ns with a weak positional restraint of 0.01 kcal/mol applied on the α-carbon (CA) atoms of the N-terminal S4-5 helices (shown as cyan spheres). (**d**) In the equilibrium simulations, the weak positional restraint in step 7 of the pre-equilibration was retained to eliminate the negative effect of removing VSD from the protein. (**e**) In some simulations (IDs 9-20 in Table B), a constraint was applied in the pre-equilibration stage (see the black frames in **b** and **c**) to sustain the interaction between Lys180/Arg180 and Glu183 residues in the outer ring. In the simulations on the DEKA mutant, the constraint was used to restrict the distance between the NZ atom of Lys180 and the δ-carbon (CD) atoms of Glu183 residues in chain A, B, C and D respectively. Therefore, the corresponding systems are denoted as DEKA-**a**, DEKA-**b**, DEKA-**c** and DEKA-**d**, respectively. The red circles highlight the constraint in various systems, where chains A, B, C and D are colored in black, red, green and blue, respectively. Similarly, in the simulations on the

DERA mutant, the constraint was applied on the ζ-carbon (CZ) atom of Arg180 and the CD atoms of Glu183 residues, and the corresponding systems are denoted as DERA-**a**, DERA-**b**, DERA-**c** and DERA-**d**, respectively.

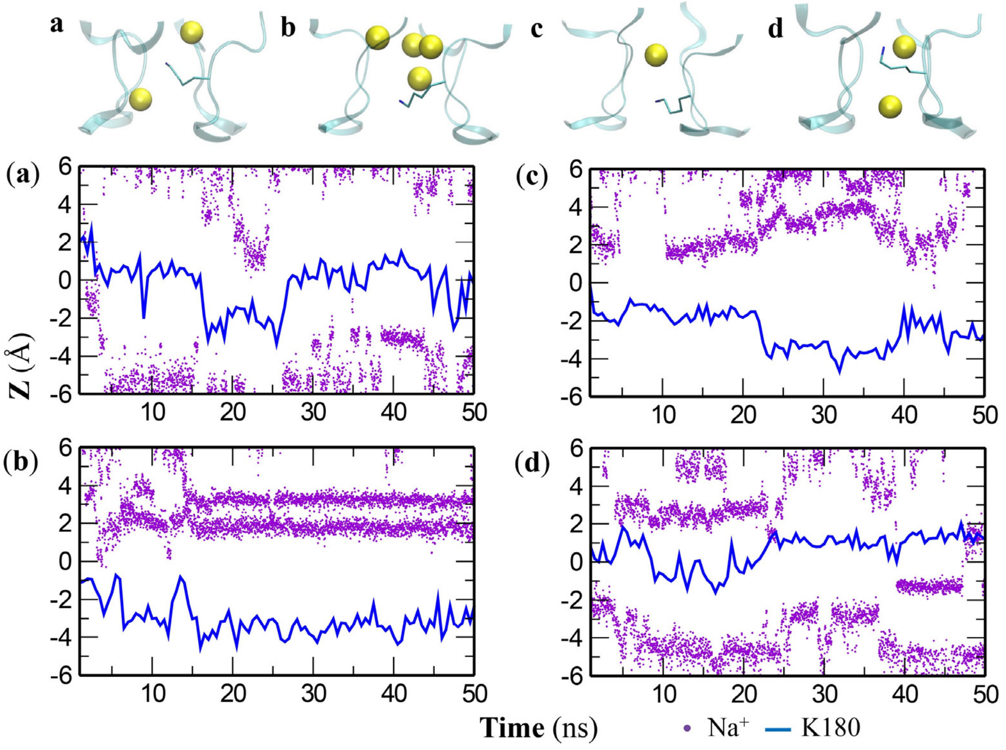

Fig D. The time-series of the vertical positions of the Na^+ ions in the DEKA-**a** (**a**), DEKA-**b** (**b**), DEKA-**c** (**c**) and DEKA-**d** (**d**) systems, Na^+ ions are represented as yellow spheres. The corresponding simulation IDs are 9, 10, 11 and 12 in Table B. The Na^+ ions can pass Lys180 side chain and arrive at $Site_{INT}$ in the DEKA-**a** and DEKA-**d** systems, while no Na^+ ions can enter the SF in the DEKA-**b** and DEKA-**c** systems. The vertical axis is the position along the z-axis, while the horizontal axis is the simulation time. The positions of the Na^+ ions and the NZ atom of Lys180 side chain are shown as violet dots and blue lines respectively.

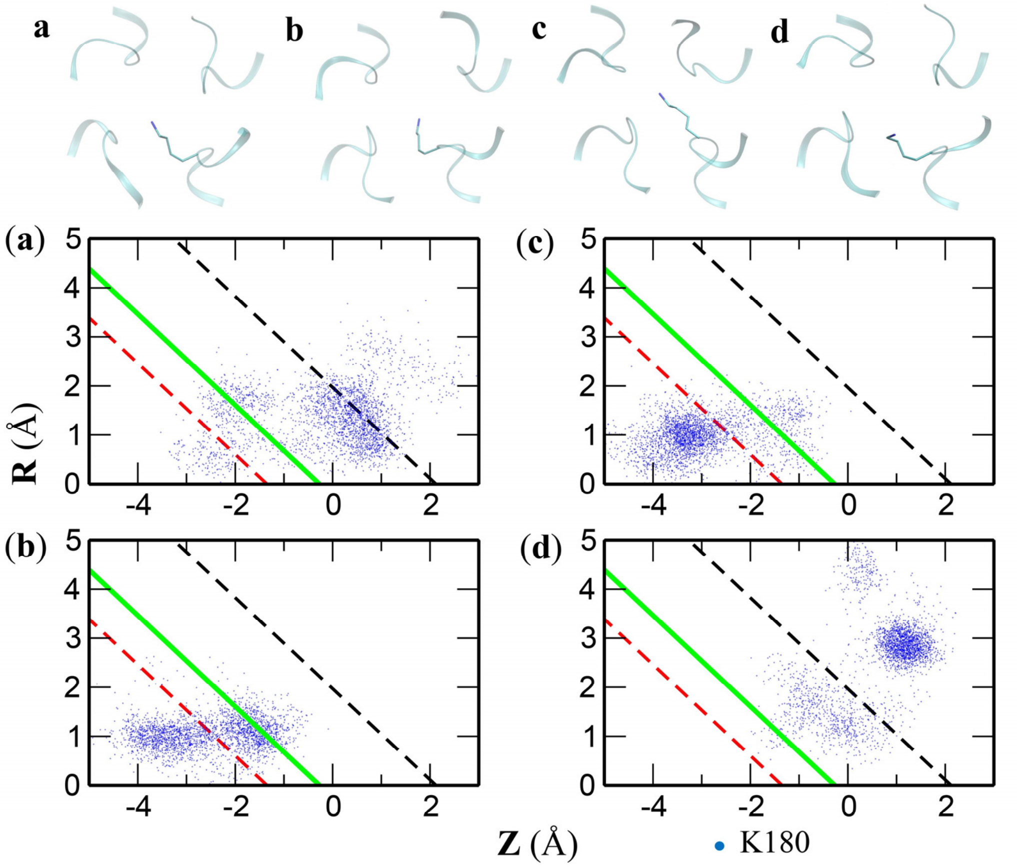

Fig E. The open and closed states of Lys 180 side chain in DEKA mutant. (**a**) In the DEKA-**a** system (ID 9 in Table B), Lys180 side chain spans over the regions of open and closed states. (**b**, **c**) In the DEKA-**b** and DEKA-**c** systems (IDs 10&11 in Table B), Lys180 side chain stays mostly in the region of the closed state. (**d**) In the DEKA-**d** system (ID 12 in Table B), Lys180 side chain stays mostly in the region of the open state. The blue dots stand for the side-chain NZ atom of Lys180. The horizontal axis (Z) is the relative position of NZ atom in the z-axis, while the vertical axis (R) is the distance between NZ atom and the center of SF in the xy-plane. Boundaries of the regions are shown in the same way as in Fig A.

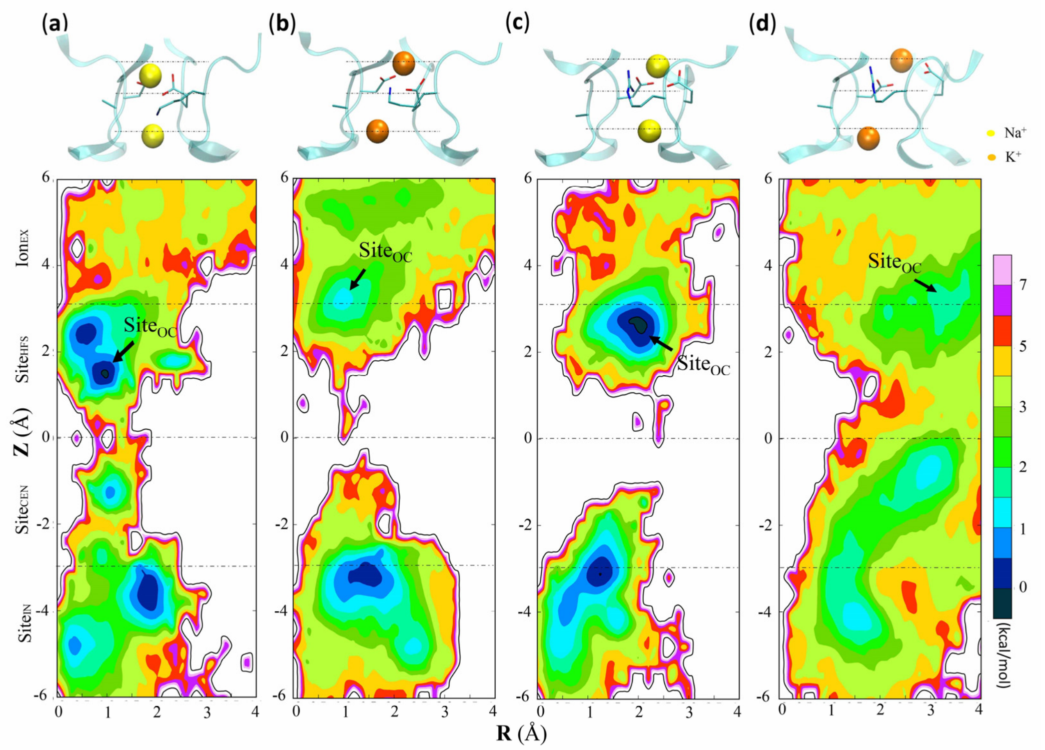

Fig F. Ion binding patterns in the SF of the DEKA (**a**, **b**) and DERA (**c**, **d**) mutants of Na_VRh for Na^+ (**a**, **c**) and K^+ (**b**, **d**) ions in the **d**-system (IDs 17-20 in Table B). For each map, the upper panel is a representative structure taken from the equilibrium simulation. The residues 180 and the overall SF are shown and colored in the same scheme as Fig 3. The lower panel is the 2D free energy profile estimated from probability density map in SF for cations. Positions of Site$_{OC}$ are labeled by black arrows. The unit of free energy as well as the vertical and horizontal axes are identical to those in Fig 3.

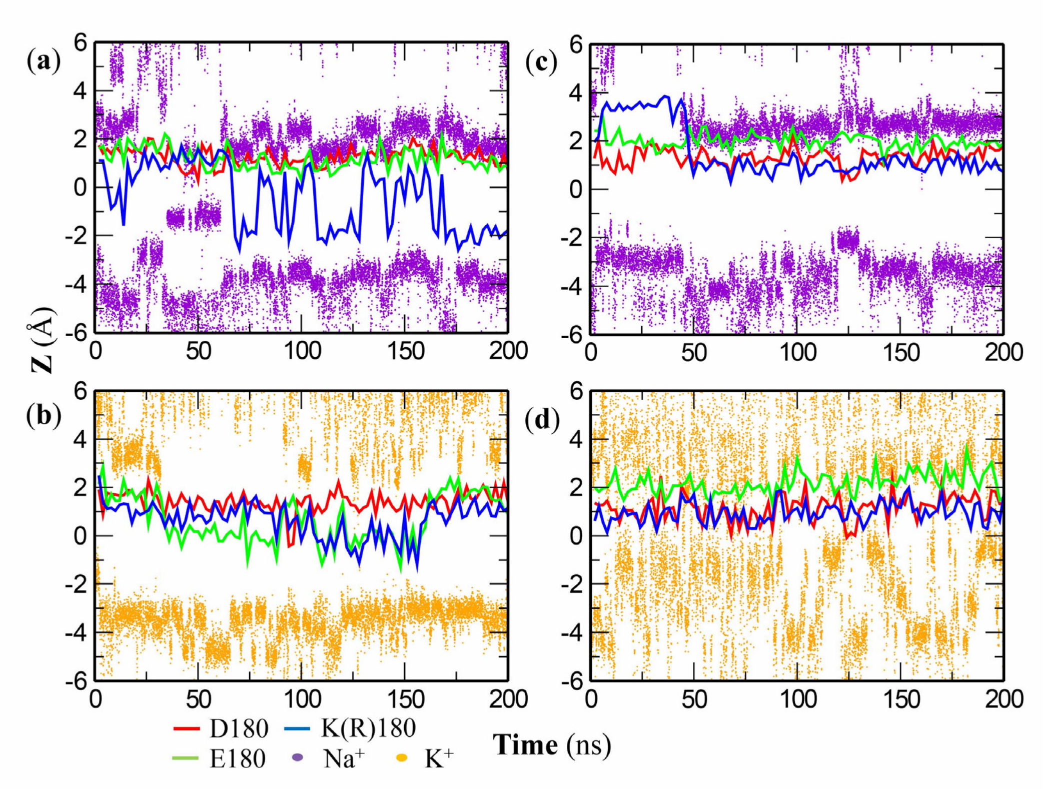

Fig G. Time-dependent ion occupancy at each binding site of the DEKA (**a**, **b**) and DERA (**c**, **d**) mutants of Na_VRh for Na^+ (**a**, **c**) and K^+ (**b**, **d**) ions in the **d**-system (IDs 17-20 in Table B). The vertical axis is the relative distance of the cation to the geometric center of the SF along the permeation pathway. Violet and orange dots are used to represent Na^+ and K^+ ions respectively. The red and green lines reflect the center of carboxylate oxygen atoms of Asp180 and Glu180 respectively. The blue line describes the position of the side-chain NZ atom of Lys180 (**a**, **b**) or the CZ atom of Arg180 (**b**, **d**).

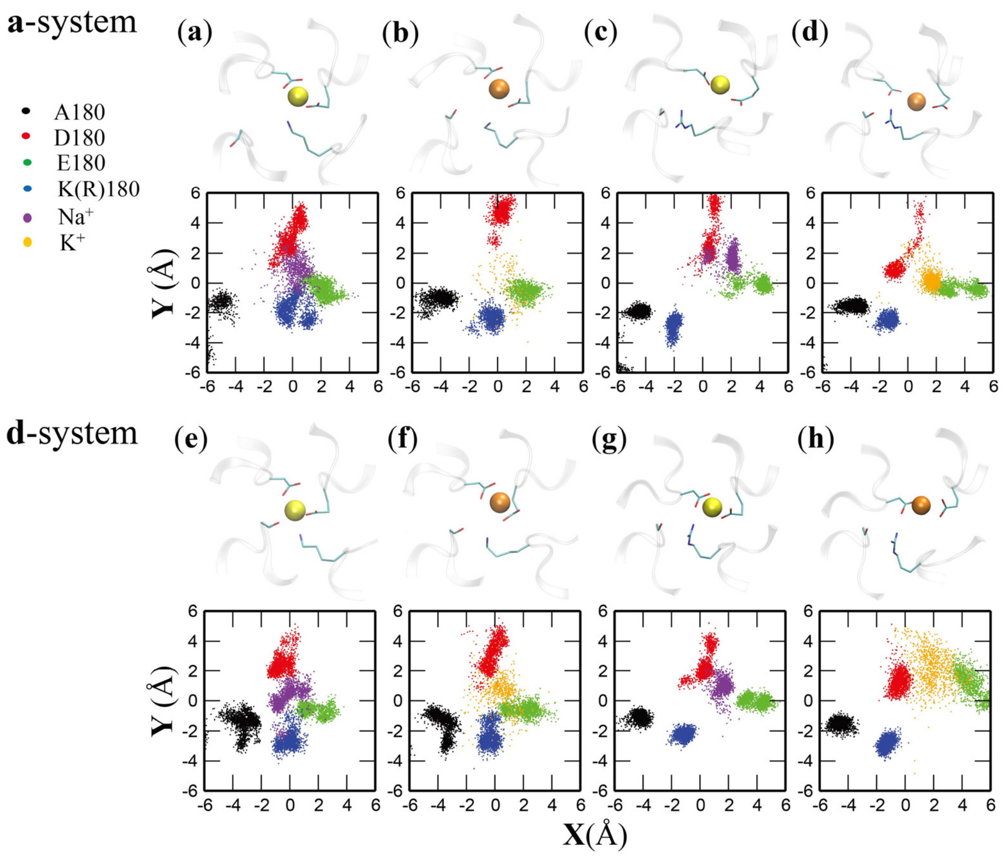

Fig H. Projections of the cations as well as side chains of residues at the constriction site in the xy-plane. Ion binding patterns of Na^+ (**a**, **c**, **e**, **g**) and K^+ (**b**, **d**, **f**, **h**) ions at $Site_{OC}$ are shown for the DEKA (**a**, **b**, **e**, **f**) and DERA (**c**, **d**, **g**, **h**) mutants of Na_VRh in the **a**-system and **d**-system (IDs 13-20 in Table B). Na^+ and K^+ ions are represented as yellow and orange spheres respectively. The black, red, green and blue dots stand for the backbone carbonyl oxygen atom of Ala180, the center of side-chain carboxylate oxygen atoms of Asp180, the center of side-chain carboxylate oxygen atoms of Glu180, and the side-chain NZ/CZ atoms of Lys180/Arg180, respectively. Violet and orange dots stand for the positions of Na^+ and K^+ ions, respectively.

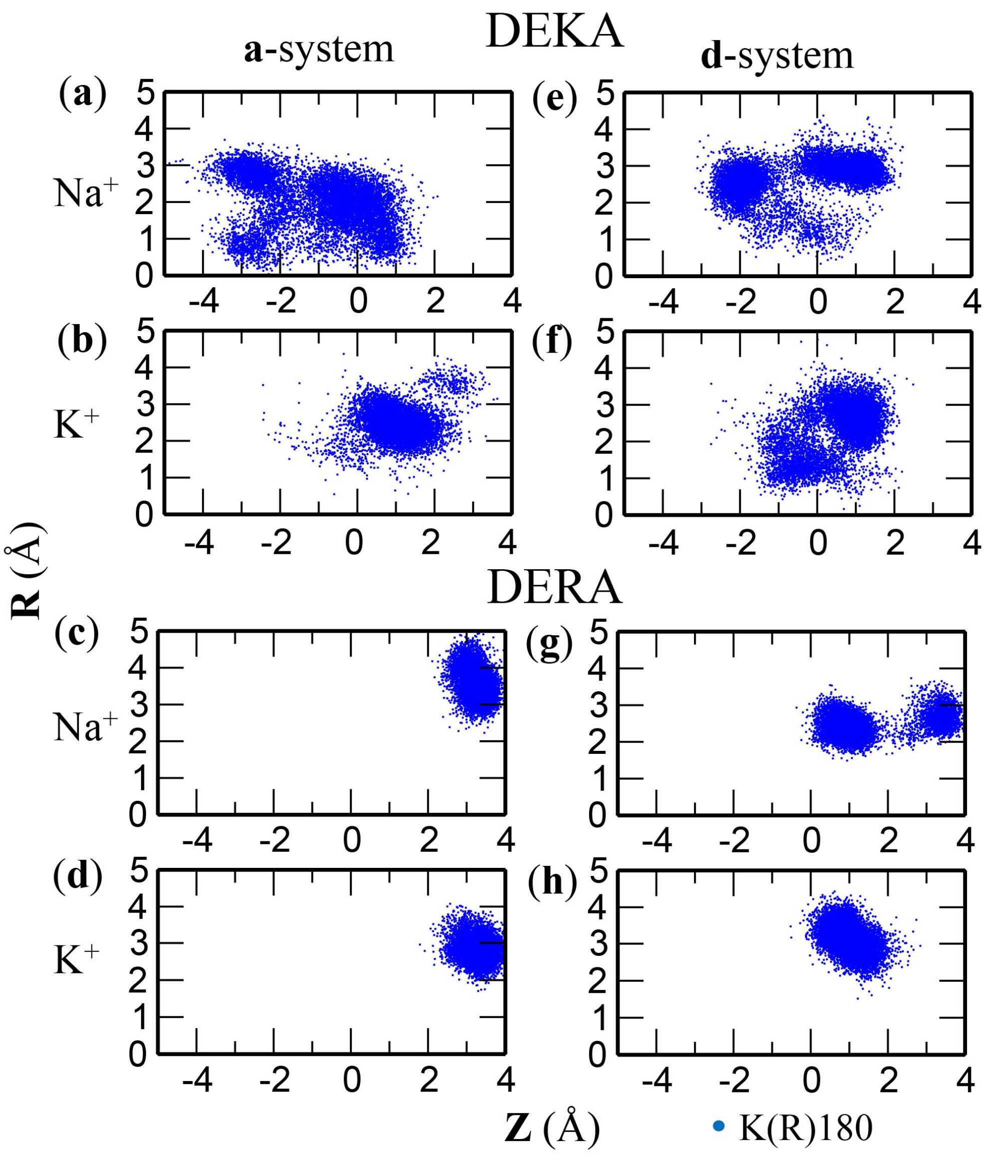

Fig I. Relative positions of the NZ/CZ atoms in the side chains of Lys180/Arg180 for the DEKA (**a**, **b**, **e**, **f**) and DERA (**c**, **d**, **g**, **h**) mutants in NaCl (**a**, **c**, **e**, **g**) and KCl (**b**, **d**, **f**, **h**) in the **a**-system (left) and **d**-system (right) (IDs 13-20 in Table B). The blue dots are chosen to show the side-chain NZ/CZ atoms of Lys180/Arg180. The horizontal axis is the relative position of NZ/CZ atom in the z-axis, while the vertical axis is the distance between NZ/CZ atom and the center of SF in the xy-plane.

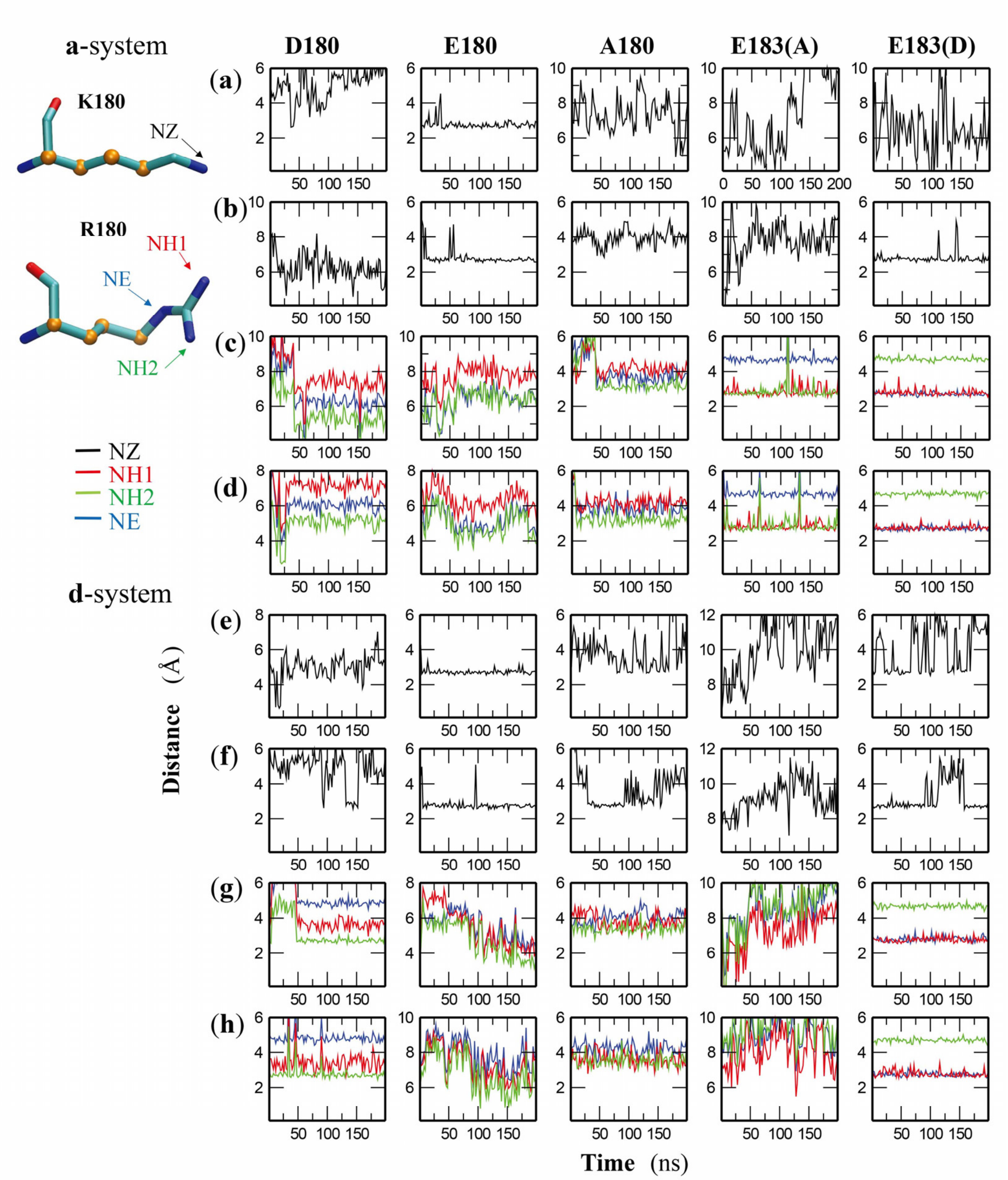

Fig J. Time-series of the minimum distances between terminal nitrogen atoms of Lys180/Arg180 and the oxygen atoms of the conserved residues, including Asp180, Glu180, Ala180, Glu183 in chain A and Glu183 in chain D, in the SF of the DEKA (**a, b, e, f**) and DERA (**c, d, g, h**) mutants of Na_VRh in NaCl (**a, c, e, g**) and KCl (**b, d, f, h**) in the **a**-system and **d**-system (IDs 13-20 in Table B). NZ stands for the side-chain amino nitrogen atom of Lys180, while NH1, NH2, and NE stand for the three side-chain guanidine nitrogen atoms of Arg180. The black line describes the

minimum distance between NZ and oxygen atoms of the other conserved residues. Red/green/blue lines reflect the minimum distance between NH1/NH2/NE and the oxygen atoms of other conserved residues. The carboxylate oxygen atoms were used for distance calculation in Asp180, Glu180, Glu183 in chain A and Glu183 in chain D, while the backbone carbonyl oxygen atom was used for Ala180.

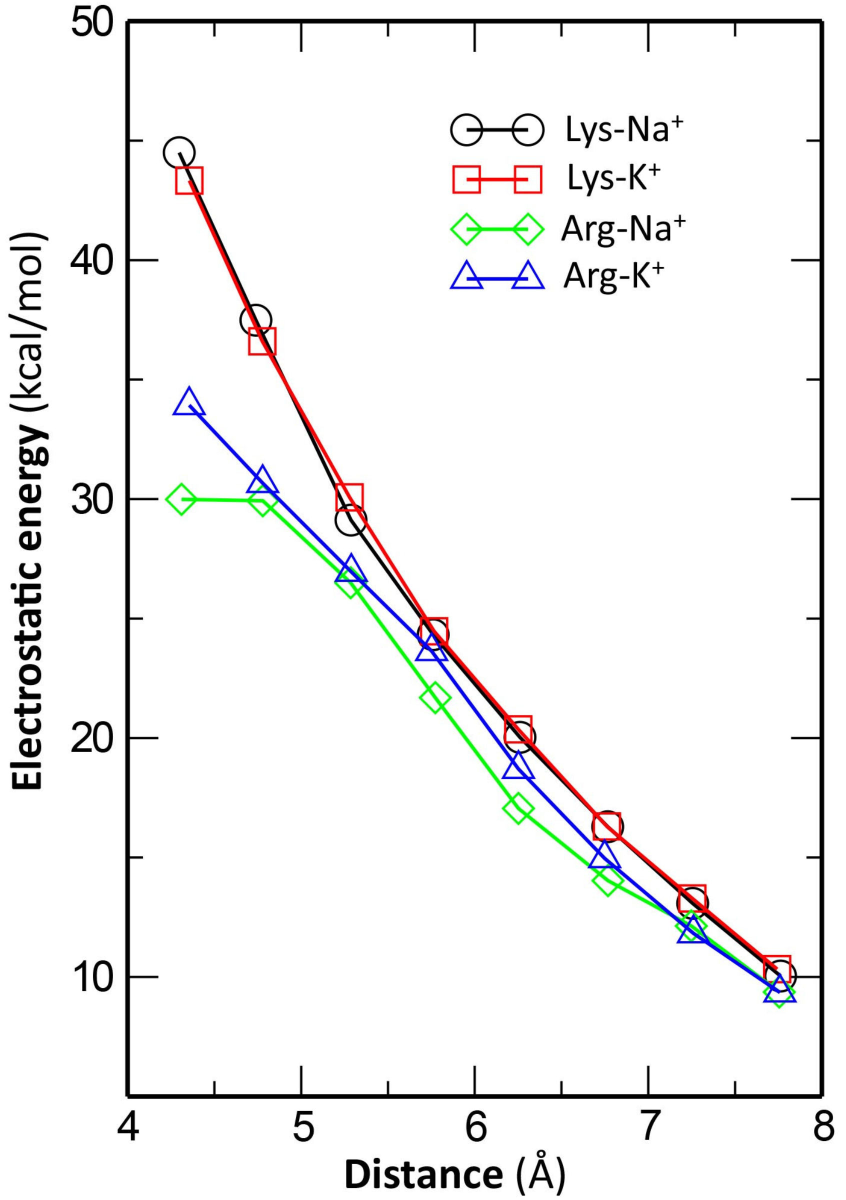

Fig K. The dependence of electrostatic repulsion on the distance between cation and the amino/guanidine group. The black circles, red squares, green diamonds and blue triangles are used to show the electrostatic interaction energies of Lys-Na^+, Lys-K^+, Arg-Na^+, and Arg-K^+ pairs at various interaction distances, respectively.

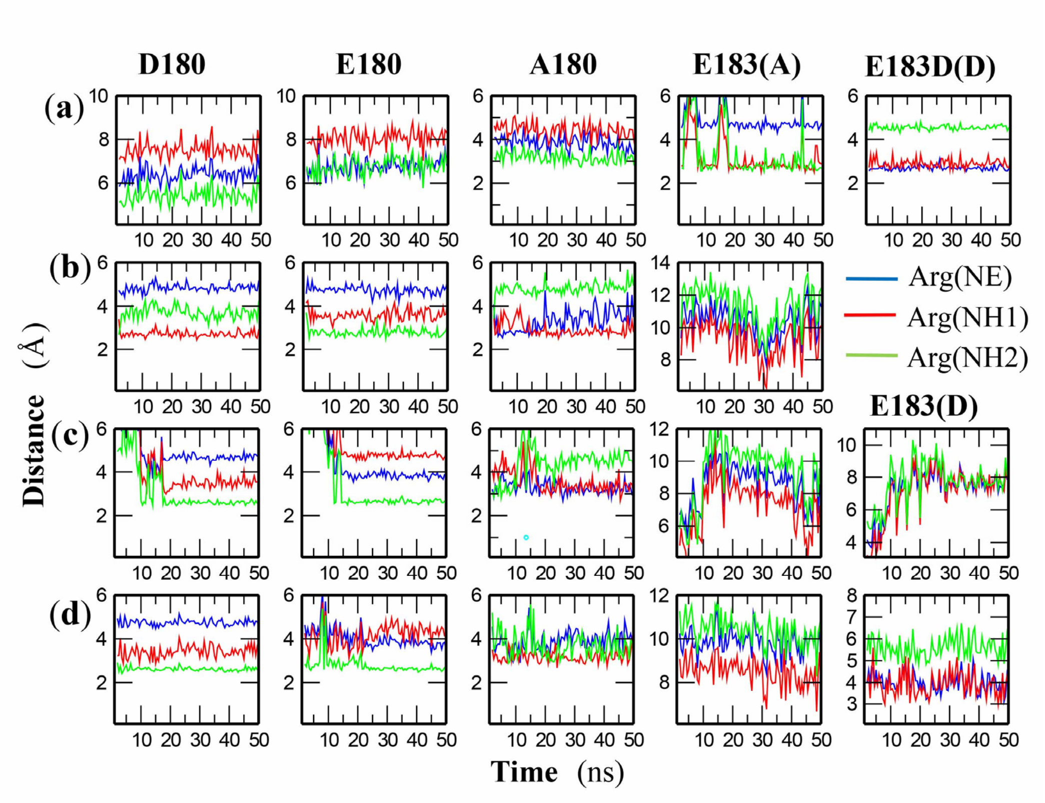

Fig L. Time-series of the minimum distances between terminal nitrogen atoms of Lys180/Arg180 and the oxygen atoms of the conserved residues in the SF of several DERA-based mutants of Na_VRh in NaCl (IDs 21, 23, 25 & 26 in Table B). Results of simulation IDs 21, 23, 25 and 26 are shown in panels **a**, **b**, **c** and **d** respectively. Representation and coloring schemes are identical to Fig J.

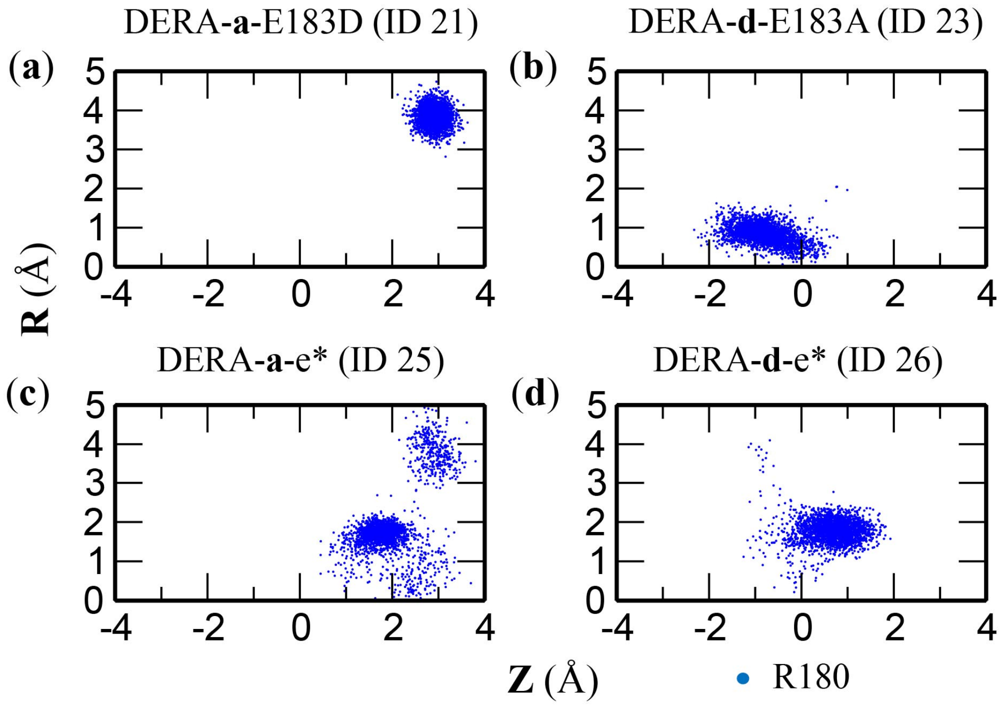

Fig M. Positions of the CZ atom of Arg180 in the simulations on the DERA-based mutants in NaCl (IDs 21, 23, 25 & 26 in Table B). The blue dots stand for the CZ atom of Arg180.

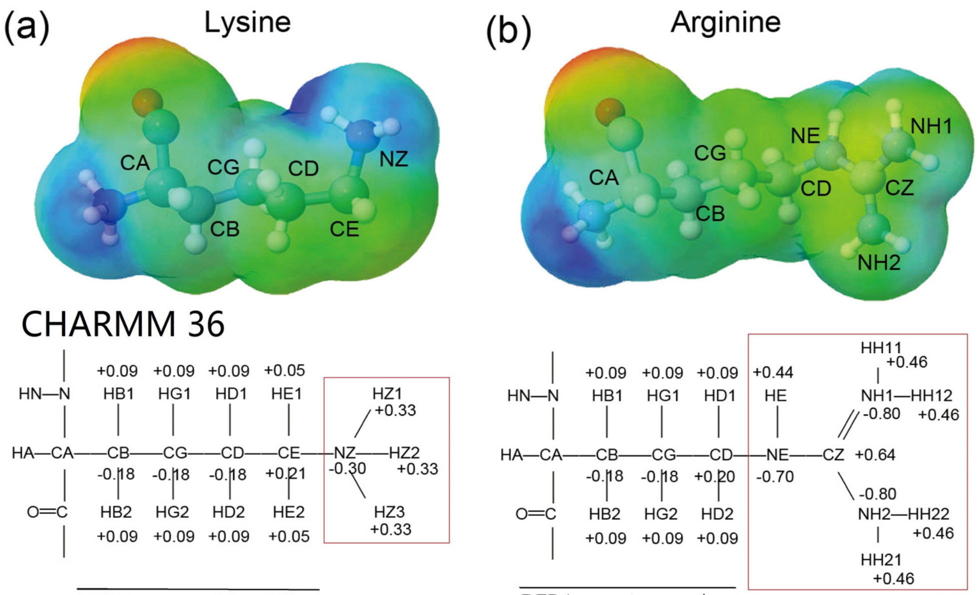

DEKA	e	0.5e	0.05e
HZ1	0.33	0.16	0.03
HZ2	0.33	0.16	0.03
HZ3	0.33	0.16	0.03

DERA	e	e*
NE	-0.7	-0.48
HE	0.44	0.04
NH1	-0.8	-0.4
NH11	0.46	0.06
NH12	0.46	0.06
NH2	-0.8	-0.22
NH21	0.46	0.46
NH22	0.46	0.46

Fig N. Distributions of positive charges in the side chains of **(a)** Lys and **(b)** Arg. The top panel shows the envelope of electrostatic potentials of Lys (**a**) and Arg (**b**) calculated using the PDB2PQR server [21]. Negative potentials are colored in red and positive ones in blue. The middle panel is the standard topologies of Lys (**a**) and Arg (**b**) in the CHARMM 36 force field [6, 8]. The red boxes highlight the amino and guanidine groups in Lys and Arg respectively. The bottom panel is the charge distributions used in equilibrium simulations on the DEKA-based (IDs 27-30 in Table B) and DERA-based (IDs 25-26 in Table B) mutants.

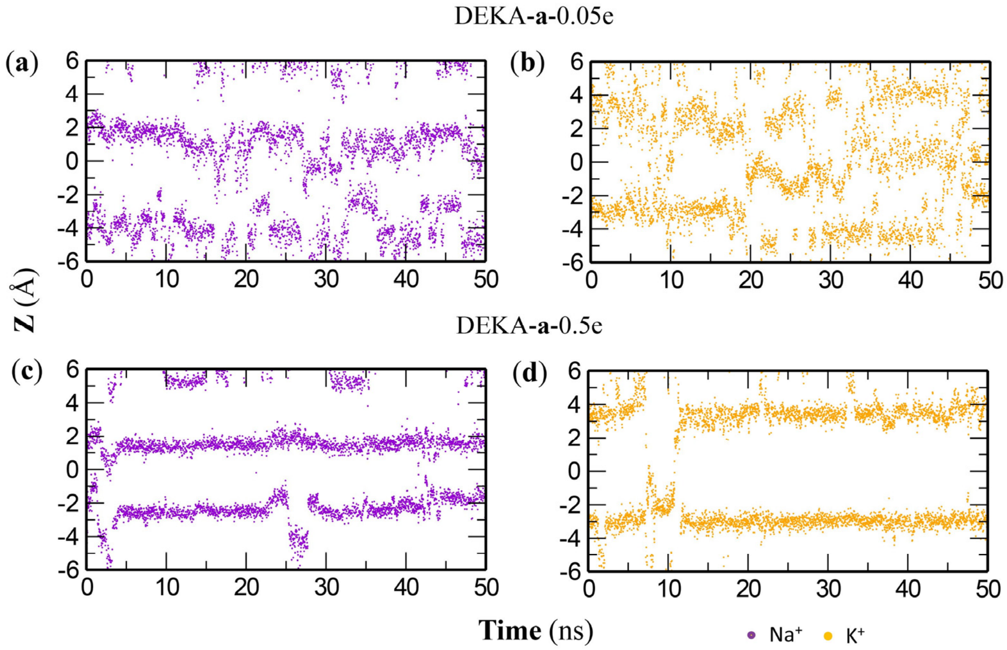

Fig O. Time-dependent ion occupancy at each binding site of the DEKA-0.05e (**a**, **b**) and DEKA-0.5e (**c**, **d**) mutants of Na_VRh for Na^+ (**a**, **c**) and K^+ (**b**, **d**) ions in the **a**-system (IDs 27-30 in Table B). The vertical axis is the relative distance of the cation to the geometric center of the SF along the permeation pathway. Violet and orange dots stand for Na^+ and K^+ ions respectively.

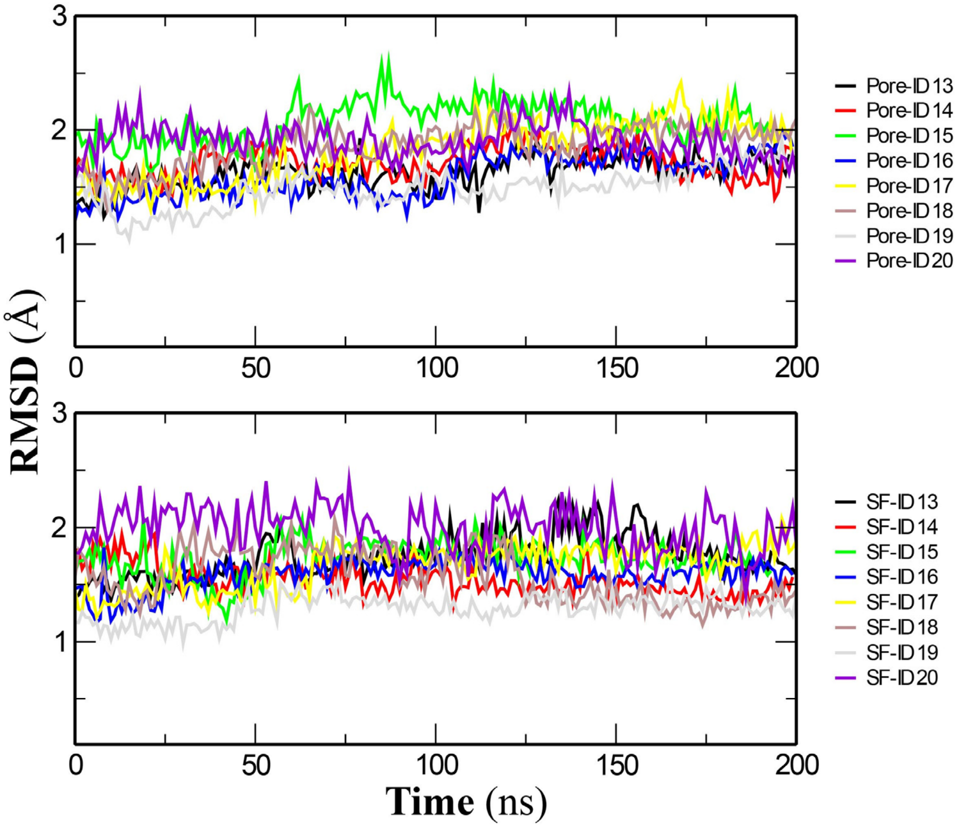

Fig P. RMSD profiles of the long equilibrium simulations (IDs 13-20 in Table B). The top and bottom panels show the RMSD profiles of the overall PD and the SF region, respectively.

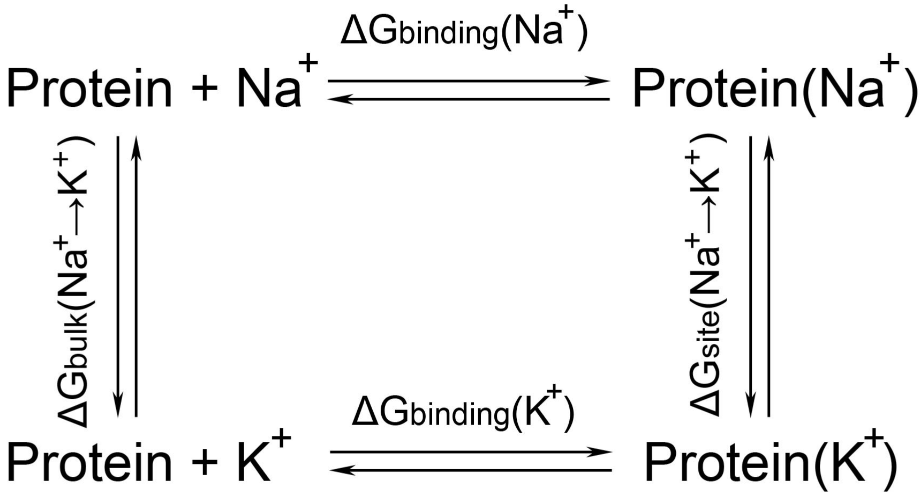

Fig Q. Thermodynamic cycle when estimating the relative binding affinity using the FEP method.

Supplementary Tables

Table A. Sequence alignment of the P-loops of the SF regions in the prokaryotic and mammalian Na_v channels.

Name	Species	DI	DII	DIII	DIV
Na_vRh	Bacteria	^{178}TLSSWET			
Na_vAb	Bacteria	^{176}TLESWSM			
Na_vMs	Bacteria	^{176}TLESWSM			
$Na_v1.1$	Rat	^{380}TQDFWEN	^{948}CGE-WIE	^{1430}TFKGWMD	^{1722}TSAGWDG
$Na_v1.4$	Human	^{404}TQDYWEN	^{759}CGE-WIE	^{1242}TFKGWMD	^{1534}TSAGWDG
$Na_v1.4a$	Zebra fish	^{371}TQDFWEN	^{763}CGE-WIE	^{1208}TFKGWMD	^{1500}TSAGWDG
$Na_v1.5$	Mouse	^{370}TQDCWER	^{898}CGE-WIE	^{1419}TFKGWMD	^{1711}TSAGWDG
$Na_v1.7$	Rabbit	^{357}TQDYWEN	^{922}CGE-WIE	^{1401}TFKGWMD	^{1693}TSAGWDG
$Na_v1.8$	Dog	^{363}TQDSWER	^{856}CGE-WIE	^{1372}TFKGWMD	^{1666}TSAGWDG
Na_v1	Fruit fly	^{386}TQDFWED	^{998}CGE-WIE	^{1507}TFKGWIQ	^{1800}TSAGWDG

DI, DII, DIII and DIV are the four structural repeats which jointly constitute the PD of Na_v channels. Only sequences of amino acid residues in the P-loops are shown with the indices of the first residues labeled in superscript. The residues located at the constriction site (inner ring) are shaded in red to show the highly conserved DEKA motif in all mammalian Na_v channels. In addition, conserved residues located at the entrance of the SF region (outer ring) are shaded in blue.

Table B. Summary of equilibrium simulations.

ID	System	Mutants (residue 180)	Ion concentration	Pre-Eq (Time) & Constraint		Eq (Time) & Constraint		First ion in SF	Other mutant & variation	Initiating structure
1	Wild-Rh	NA	150mM NaCl	10 ns	No	50 ns	No	Site$_{INT}$	NA	4DXW
2	Wild-Rh	NA	150mM KCl	10 ns	No	50 ns	No	Site$_{INT}$	NA	4DXW
3	DEAA	DEAA	150mM NaCl	10 ns	No	50 ns	No	Site$_{INT}$	NA	4DXW*
4	DEAA	DEAA	150mM KCl	10 ns	No	50 ns	No	Site$_{INT}$	NA	4DXW*
5	DEKA	DEKA	150mM NaCl	10 ns	No	50 ns	No	Site$_{OC}$	NA	4DXW*
6	DEKA	DEKA	150mM NaCl	10 ns	No	50 ns	No	Site$_{INT}$	NA	4DXW*
7	DEKA	DEKA	150mM KCl	10 ns	No	50 ns	No	Site$_{OC}$	NA	4DXW*
8	DERA	DERA	150mM NaCl	10 ns	No	50 ns	No	Site$_{OC}$	NA	4DXW*
9	DEKA-**a**	DEKA	150mM NaCl	20 ns	Yes	50 ns	No	Site$_{INT}$	NA	4DXW*
10	DEKA-**b**	DEKA	150mM NaCl	20 ns	Yes	50 ns	No	Site$_{OC}$	NA	4DXW*
11	DEKA-**c**	DEKA	150mM NaCl	20 ns	Yes	50 ns	No	Site$_{OC}$	NA	4DXW*
12	DEKA-**d**	DEKA	150mM NaCl	20 ns	Yes	50 ns	No	Site$_{INT}$	NA	4DXW*
13	DEKA-**a**	DEKA	150mM NaCl	20 ns	Yes	200 ns	No	Site$_{INT}$	NA	4DXW*
14	DEKA-**a**	DEKA	150mM KCl	20 ns	Yes	200 ns	No	Site$_{INT}$	NA	4DXW*

15	DERA-**a**	DERA	150mM NaCl	20 ns	Yes	200 ns	No	$Site_{INT}$	NA	4DXW*
16	DERA-**a**	DERA	150mM KCl	20 ns	Yes	200 ns	No	$Site_{INT}$	NA	4DXW*
17	DEKA-**d**	DEKA	150mM NaCl	20 ns	Yes	200 ns	No	$Site_{INT}$	NA	4DXW*
18	DEKA-**d**	DEKA	150mM KCl	20 ns	Yes	200 ns	No	$Site_{INT}$	NA	4DXW*
19	DERA-**d**	DERA	150mM NaCl	20 ns	Yes	200 ns	No	$Site_{INT}$	NA	4DXW*
20	DERA-**d**	DERA	150mM KCl	20 ns	Yes	200 ns	No	$Site_{INT}$	NA	4DXW*
21	DERA-**a**-E183D	DERA	150mM NaCl	1 ns	No	50 ns	No	NA	E183D(A)	ID 15, cluster 1
22	DERA-**a**-E183D	DERA	150mM KCl	1 ns	No	50 ns	No	NA	E183D(A)	ID 16, cluster 1
23	DERA-**d**-E183A	DERA	150mM NaCl	1 ns	No	50 ns	No	NA	E183A(D)	ID 19, cluster 1
24	DERA-**d**-E183A	DERA	150mM KCl	1 ns	No	50 ns	No	NA	E183A(D)	ID 20, cluster 1
25	DERA-**a**-e*	DERA	150mM NaCl	1 ns	No	50 ns	No	NA	R180(e*)	ID 15, cluster 1
26	DERA-**d**-e*	DERA	150mM NaCl	1 ns	No	50 ns	No	NA	R180(e*)	ID 19, cluster 1
27	DEKA-**a**-0.05e	DEKA	150mM NaCl	1 ns	No	50 ns	No	NA	K180(0.05e)	ID 13, cluster 1
28	DEKA-**a**-0.05e	DEKA	150mM KCl	1 ns	No	50 ns	No	NA	K180(0.05e)	ID 14, cluster 1
29	DEKA-**a**-0.5e	DEKA	150mM NaCl	1 ns	No	50 ns	No	NA	K180(0.5e)	ID 13, cluster 1
30	DEKA-**a**-0.5e	DEKA	150mM KCl	1 ns	No	50 ns	No	NA	K180(0.5e)	ID 14, cluster 1

4DXW* means that the structure was created by mutation from the crystal structure of 4DXW. NA stands for “not applicable”. 0.05e, 0.5e, and e* stand for artificially modified charge density on Arg180/Lys180 (see details in Fig N).

Table C. Analysis on states of Lys180/Arg180 in the SF.

ID	System	Ion type	State percentage of Lys180/Arg180 (%)		
			Closed	Intermediate	Open
5	DEKA	Na^+	84.76	15.24	0
6	DEKA	Na^+	38.44	33.45	28.11
7	DEKA	K^+	61.25	38.75	0
8	DERA	Na^+	98.40	1.60	0
9	DEKA-**a**	Na^+	6.30	67.22	26.48
10	DEKA-**b**	Na^+	76.59	23.41	0
11	DEKA-**c**	Na^+	50.85	49.15	0
12	DEKA-**d**	Na^+	0	26.34	73.66
13	DEKA-**a**	Na^+	6.95	70.63	22.42
14	DEKA-**a**	K^+	0	2.45	97.55
15	DERA-**a**	Na^+	0	0	100
16	DERA-**a**	K^+	0	0	100
17	DEKA-**d**	Na^+	0	49.91	50.09
18	DEKA-**d**	K^+	0	22.91	77.09
19	DERA-**d**	Na^+	0	0	100
20	DERA-**d**	K^+	0	0	100
21	DERA-**a**-E183D	Na^+	0	0	100
22	DERA-**a**-E183D	K^+	0	0	100
23	DERA-**d**-E183A	Na^+	0	100	0
24	DERA-**d**-E183A	K^+	5.88	48.16	45.96
25	DERA-**a**-e*	Na^+	0	1.32	98.68
26	DERA-**d**-e*	Na^+	0	16.00	84.00

Table D. Analysis on ion occupancy in the SF.

ID	System	Ion type	Ion occupancy in the SF (%)	
			One ion	Two ions
13	DEKA-**a**	Na^+	51.12	48.45
14	DEKA-**a**	K^+	99.01	0.98
15	DERA-**a**	Na^+	17.48	82.51
16	DERA-**a**	K^+	8.62	91.37
17	DEKA-**d**	Na^+	7.44	92.55
18	DEKA-**d**	K^+	84.75	15.24
19	DERA-**d**	Na^+	26.74	73.25
20	DERA-**d**	K^+	65.87	33.61
27	DEKA-**a**-0.05e	Na^+	3.64	78.28
28	DEKA-**a**-0.05e	K^+	15.76	84.20
29	DEKA-**a**-0.5e	Na^+	23.40	76.56
30	DEKA-**a**-0.5e	K^+	25.60	74.35

Table E. Analysis on the important hydrogen bonds in the SF.

ID	System	Ion type	Hydrogen bonds donor	Hydrogen bonds acceptor (%)				
				D180	E180	A180	E183(A)	E183(D)
13	DEKA-**a**	Na^+	K180	2.15	51.37	0	0	0.21
14	DEKA-**a**	K^+	K180	0	49.14	0.41	0.88	36.96
15	DERA-**a**	Na^+	R180	0	0	0	67.89(22.57)	91.50(54.56)
16	DERA-**a**	K^+	R180	0	0	0	67.47(22.88)	92.32(52.52)
17	DEKA-**d**	Na^+	K180	0.92	52.75	8.39	0	19.06
18	DEKA-**d**	K^+	K180	2.15	50.69	12.60	0	35.05
19	DERA-**d**	Na^+	R180	52.08(0.01)		0	1.25(0.33)	82.02(41.21)
20	DERA-**d**	K^+	R180	63.02(0.15)		0	0	89.36(50.42)
21	DERA-**a**-E183D	Na^+	R180	0	0	0	45.28(26.24)	38.76(8.60)
22	DERA-**a**-E183D	K^+	R180	0	0	0	41.48(18.00)	47.00(1.76)
23	DERA-**d**-E183A	Na^+	R180	42.88(5.80)	7.12	7.68	0	0
24	DERA-**d**-E183A	K^+	R180	51.52	36.88	47.28	0	0

The hydrogen bonds between the side chains of Lys180/Arg180 and several conserved residues in the DEKA and DERA mutants, are calculated

for the percentage of frames forming at least one hydrogen bond and that forming at least two hydrogen bonds (in parenthesis) from the equilibrium trajectories.

Table F. Main cation binding modes in the DEKA and DERA mutants.

ID	System	Ion type	Ion Occupancy	Binding site	Cluster No.	Cluster frames	Percentage (%)
13	DEKA-**a**	Na^+	5328 (7500)	$Site_{OC}$	1	681(5328)	12.78
					2	366(5328)	6.86
					3	212(5328)	3.94
14	DEKA-**a**	K^+	846 (7500)	$Site_{OC}$	1	70(846)	8.27
					2	68(846)	8.03
					3	62(846)	7.32
15	DERA-**a**	Na^+	7150 (7500)	$Site_{OC}$	1	3042(7150)	42.54
					2	2149(7150)	30.05
					3	457(7150)	6.39
16	DERA-**a**	K^+	7500 (7500)	$Site_{OC}$	1	4109(7500)	54.78
					2	1010(7500)	13.46
					3	652(7500)	8.69
17	DEKA-**d**	Na^+	7500 (7500)	$Site_{OC}$	1	2034(7500)	27.12
					2	1266(7500)	16.88
					3	517(7500)	6.89
18	DEKA-**d**	K^+	1817 (7500)	$Site_{OC}$	1	143(1817)	7.87
					2	141(1817)	7.76
					3	87(1817)	4.78
19	DERA-**d**	Na^+	7500 (7500)	$Site_{OC}$	1	2282(7500)	30.42
					2	1606(7500)	21.41
					3	1247(7500)	16.62
20	DERA-**d**	K^+	3891 (7500)	$Site_{OC}$	1	158(3891)	4.06
					2	101(3891)	2.59
					3	94(3891)	2.41

Numbers in the parenthesis on the right side stand for the maximum numbers of cation-bound frames in the simulations.

Table G. Distances between cation and changed residues in the long equilibrium simulations.

ID	System	Ion type	Binding site	Cluster No.	The distance between cation and specific atom					$\Delta\Delta G(Na^{+}\rightarrow k^{+})$ (kcal/mol)	# of well-coordinated
					OD1	OD2	OE1	OE2	N_{MIN}		
13	DEKA-**a**	Na^+	$Site_{OC}$	1	2.28±0.10	2.36±0.16	2.48±0.21	2.27±0.10	3.85±0.26	2.56±0.40	4
				2	2.39±0.17	2.33±0.13	2.37±0.36	**4.04±0.27**	6.64±0.26	1.17±0.25	3
14	DEKA-**a**	K^+	$Site_{OC}$	1	2.78±0.22	2.72±0.14	2.69±0.22	**3.28±0.44**	4.48±0.45	1.57±0.34	3
15	DERA-**a**	Na^+	$Site_{OC}$	1	2.34±0.12	2.32±0.13	2.25±0.10	**3.72±0.30**	5.67±0.37	3.84±0.60	3
				2	2.35±0.13	2.32±0.12	**3.73±0.31**	2.27±0.12	5.65±0.35	3.66±0.21	3
16	DERA-**a**	K^+	$Site_{OC}$	1	**3.30±0.30**	2.55±0.10	**4.59±0.14**	2.57±0.11	6.13±0.22	-0.46±0.43	2
				2	**3.56±0.28**	2.56±0.10	**6.98±0.28**	**5.01±0.29**	6.11±0.36	0.09±0.83	1
17	DEKA-**d**	Na^+	$Site_{OC}$	1	**3.14±0.51**	2.25±0.10	2.31±0.11	2.39±0.16	4.26±0.23	2.84±0.31	3
				2	2.42±0.20	2.26±0.09	2.57±0.33	2.28±0.11	4.05±0.27	3.80±0.17	4
18	DEKA-**d**	K^+	$Site_{OC}$	1	2.68±0.13	2.78±0.19	2.88±0.25	2.78±0.20	4.46±0.31	3.45±0.63	4
19	DERA-**d**	Na^+	$Site_{OC}$	1	2.33±0.12	2.32±0.11	2.50±0.37	2.30±0.13	3.91±0.25	3.35±0.20	4
				2	2.35±0.16	2.31±0.11	2.29±0.11	2.42±0.22	3.83±0.25	3.94±0.21	4
20	DERA-**d**	K^+	$Site_{OC}$	1	2.62±0.16	**3.32±0.48**	2.93±0.39	2.70±0.21	5.26±0.64	2.39±0.17	3
				2	2.62±0.17	**3.30±0.45**	2.67±0.17	2.83±0.28	4.96±0.56	2.32±0.16	3

OD1 and OD2 are the two carboxylate oxygen atoms of Asp180, while OE1 and OE2 are the two carboxylate oxygen atoms of Glu180. N_{MIN} is the minimum distance between cation and the side-chain nitrogen atoms of Lys180/Arg180. The distances between cations and oxygen atoms are shown in bold to indicate the lack of good coordination, as long as the mean value is smaller than the cutoff one (2.69 Å and 3.22 Å for Na^+ and K^+ ions respectively).

Table H. Analysis on the distances between cation and several protein oxygen atoms.

ID	System	Ion type	Binding site	Cluster No.	The distance between ion with other atom		# of well-coordinated
					OA	OS_{MIN}	
16	DERA-**a**	K^+	$Site_{OC}$	1	6.39±0.47	5.68±0.82	0
				2	6.45±0.45	5.59±0.95	0

OA is the minimum distance between cation and the backbone carbonyl oxygen atom of Ala180. OS_{MIN} is the minimum distance between cation and hydroxyl oxygen atoms of four Ser181 residues.

Table I. Estimating the reference free energy change in bulk water in the FEP simulation.

Repeats	Forward (kcal/mol)	Backward (kcal/mol)	BAR estimator (kcal/mol)	Average $\Delta G(Na^{+} \rightarrow K^{+})$ (kcal/mol)
1	18.5164	-18.4799	18.50±0.05	
2	18.3288	-18.4581	18.41±0.05	18.47±0.05
3	18.4596	-18.5225	18.50±0.05	

BAR is the Bennett acceptance ratio estimator of free energy in the FEP calculation.

Table K. Details of FEP calculations.

Simulation ID	Cluster	Forward (kcal/mol)	Backward (kcal/mol)	BAR estimator (kcal/mol)	Average $\Delta G(Na^{+}\rightarrow K^{+})$ (kcal/mol)	$\Delta\Delta G(Na^{+}\rightarrow K^{+})$ (kcal/mol)
13	1	21.3672	-19.7885	20.59±0.06	21.03±0.40	2.56±0.40
		22.1968	-20.0059	21.14±0.05		
		22.2115	-20.5401	21.37±0.07		
	2	20.2311	-18.4729	19.36±0.06	19.64±0.25	1.17±0.25
		19.4809	-19.917	19.72±0.06		
		19.9656	-19.7193	19.84±0.05		
14	1	-20.5816	20.0856	-20.35±0.10	20.04±0.34	1.57±0.34
		-19.783	20.3901	-20.11±0.10		
		-19.7567	19.6015	-19.67±0.11		
15	1	22.0965	-23.5976	22.88±0.05	22.31±0.60	3.84±0.60
		22.6931	-21.9818	22.36±0.06		
		22.5486	-20.8095	21.69±0.05		
	2	22.3319	-22.2676	22.27±0.06	22.13±0.21	3.66±0.22
		22.672	-21.7728	22.23±0.06		
		21.981	-21.7497	21.88±0.06		
16	1	-16.91	18.1439	-17.54±0.10	18.01±0.43	-0.46±0.43
		-18.3128	18.4533	-18.39±0.09		
		-17.6892	18.5624	-18.11±0.09		
	2	-17.8838	20.0101	-18.91±0.08	18.56±0.83	0.09±0.83
		-17.6247	17.6382	-17.61±0.10		
		-18.2111	20.1738	-19.15±0.06		
17	1	21.4048	-20.8089	21.10±0.05	21.31±0.31	2.84±0.31
		21.2644	-21.0767	21.16±0.06		
		22.1096	-21.2159	21.67±0.05		

	2	22.3003	-22.3079	22.31±0.06	22.27±0.17	3.80±0.18
		21.928	-22.224	22.08±0.06		
		23.0807	-21.7252	22.42±0.06		
18	1	-20.8941	22.1562	-21.53±0.09	21.92±0.63	3.45±0.63
		-22.6356	22.6278	-22.65±0.10		
		-21.7067	21.4058	-21.59±0.10		
19	1	22.2924	-21.2235	21.73±0.06	21.82±0.20	3.35±0.21
		22.5446	-21.5915	22.05±0.06		
		22.4556	-20.9185	21.69±0.06		
	2	22.3317	-22.1397	22.26±0.05	22.41±0.21	3.94±0.22
		22.6006	-22.6849	22.65±0.05		
		23.0788	-21.597	22.32±0.06		
20	1	-20.9832	21.0099	-20.99±0.09	20.86±0.17	2.39±0.18
		-20.7289	21.1573	-20.92±0.09		
		-20.5828	20.6951	-20.67±0.10		
	2	-20.2526	20.9339	-20.62±0.09	20.79±0.16	2.32±0.17
		-20.8095	20.8136	-20.82±0.09		
		-20.7287	21.1611	-20.93±0.09		

The reference value, $\Delta G_{bulk}(Na^{+}\rightarrow K^{+})$ = 18.47 ± 0.05 kcal/mol, was calculated in Table I, and was subtracted from the $\Delta G(Na^{+}\rightarrow K^{+})$ to derive the relative binding affinity $\Delta\Delta G(Na^{+}\rightarrow K^{+})$.

Supplementary References

1. Xia M, Liu H, Li Y, Yan N, Gong H. The mechanism of Na(+)/K(+) selectivity in mammalian voltage-gated sodium channels based on molecular dynamics simulation. Biophys J. 2013;104(11):2401-9. doi: 10.1016/j.bpj.2013.04.035. PubMed PMID: 23746512; PubMed Central PMCID: PMC3672897.
2. Humphrey W, Dalke A, Schulten K. VMD: Visual molecular dynamics. Journal of Molecular Graphics. 1996;14(1):33-8. doi: 10.1016/0263-7855(96)00018-5.
3. Phillips JC, Braun R, Wang W, Gumbart J, Tajkhorshid E, Villa E, et al. Scalable molecular dynamics with NAMD. J Comput Chem. 2005;26(16):1781-802. doi: 10.1002/jcc.20289.
4. Martyna GJ, Tobias DJ, Klein ML. Constant pressure molecular dynamics algorithms. The Journal of Chemical Physics. 1994;101(5):4177-89.
5. Feller SE, Zhang Y, Pastor RW, Brooks BR. Constant pressure molecular dynamics simulation: The Langevin piston method. The Journal of Chemical Physics. 1995;103(11):4613-21.
6. Mackerell AD, Jr., Feig M, Brooks CL, 3rd. Extending the treatment of backbone energetics in protein force fields: limitations of gas-phase quantum mechanics in reproducing protein conformational distributions in molecular dynamics simulations. J Comput Chem. 2004;25(11):1400-15. Epub 2004/06/09. doi: 10.1002/jcc.20065. PubMed PMID: 15185334.
7. Feller SE, MacKerell AD. An Improved Empirical Potential Energy Function for Molecular Simulations of Phospholipids. The Journal of Physical Chemistry B. 2000;104(31):7510-5. doi: 10.1021/jp0007843.
8. MacKerell AD, Bashford D, Bellott, Dunbrack RL, Evanseck JD, Field MJ, et al. All-Atom Empirical Potential for Molecular Modeling and Dynamics Studies of Proteins†. The Journal of Physical Chemistry B. 1998;102(18):3586-616. doi: 10.1021/jp973084f.
9. Noskov SY, Berneche S, Roux B. Control of ion selectivity in potassium channels by electrostatic and dynamic properties of carbonyl ligands. Nature. 2004;431(7010):830-4. doi: http://www.nature.com/nature/journal/v431/n7010/suppinfo/nature02943_S1.html.
10. Essmann U, Perera L, Berkowitz M, Darden T, Lee H, Pedersen L. A smooth particle mesh Ewald method. The Journal of Chemical Physics. 1995;103(19):8577-93. doi: citeulike-article-id:487557.
11. Miyamoto S, Kollman P. Settle: An analytical version of the SHAKE and RATTLE algorithm for rigid water models. Journal of Computational Chemistry. 1992;13(8):952-62. doi: citeulike-article-id:3740710.
12. Zwanzig RW. High-Temperature Equation of State by a Perturbation Method. II. Polar Gases. The Journal of Chemical Physics. 1955;23(10):1915-22.
13. Kollman P. Free energy calculations: Applications to chemical and biochemical phenomena. Chemical Reviews. 1993;93(7):2395-417. doi: 10.1021/cr00023a004.
14. Bennett CH. Efficient estimation of free energy differences from Monte Carlo data. Journal of Computational Physics. 1976;22(2):245-68. doi: 10.1016/0021-9991(76)90078-4.
15. Pohorille A, Jarzynski C, Chipot C. Good Practices in Free-Energy Calculations. The Journal of Physical Chemistry B. 2010;114(32):10235-53. doi: 10.1021/jp102971x.
16. Gao J, Kuczera K, Tidor B, Karplus M. Hidden thermodynamics of mutant proteins: a molecular dynamics analysis. Science. 1989;244(4908):1069-72. Epub 1989/06/02. PubMed PMID: 2727695.
17. Pearlman DA. A Comparison of Alternative Approaches to Free Energy Calculations. The Journal of Physical Chemistry. 1994;98(5):1487-93. doi: 10.1021/j100056a020.

18. Zacharias M, Straatsma TP, McCammon JA. Separation-shifted scaling, a new scaling method for Lennard-Jones interactions in thermodynamic integration. J Chem Phys. 1994;100:9025-31.
19. Marcus Y. Thermodynamics of solvation of ions. Part 5.-Gibbs free energy of hydration at 298.15 K. Journal of the Chemical Society, Faraday Transactions. 1991;87(18):2995-9.
20. Schmid R, Miah AM, Sapunov VN. A new table of the thermodynamic quantities of ionic hydration: values and some applications (enthalpy-entropy compensation and Born radii). Physical Chemistry Chemical Physics. 2000;2(1):97-102.
21. Dolinsky TJ, Nielsen JE, McCammon JA, Baker NA. PDB2PQR: an automated pipeline for the setup of Poisson-Boltzmann electrostatics calculations. Nucleic Acids Res. 2004;32(Web Server issue):W665-7. Epub 2004/06/25. doi: 10.1093/nar/gkh381
32/suppl_2/W665 [pii]. PubMed PMID: 15215472; PubMed Central PMCID: PMC441519.
